# Bricks, trusses and superstructures: Strategies for skeletal reinforcement in batoid fishes (rays and skates)

**DOI:** 10.3389/fcell.2022.932341

**Published:** 2022-10-12

**Authors:** Brett Clark, Júlia Chaumel, Zerina Johanson, Charlie Underwood, Moya M. Smith, Mason N. Dean

**Affiliations:** ^1^ Image and Analysis Centre, Core Research Labs, London, United Kingdom; ^2^ Department of Biomaterials, Max Planck Institute of Colloids and Interfaces, Potsdam, Germany; ^3^ Natural History Museum, London, United Kingdom; ^4^ Department of Earth and Planetary Sciences, Birkbeck, University of London, London, United Kingdom; ^5^ Centre for Craniofacial and Regenerative Biology, Dental Institute, King’s College, London, United Kingdom; ^6^ Department of Infectious Diseases and Public Health, City University of Hong Kong, Kowloon Tong, Hong Kong SAR, China

**Keywords:** Batoidea, durophagy, jaw, trabeculae, tesserae, tessellated cartilage, skeletal biomaterials

## Abstract

Crushing and eating hard prey (durophagy) is mechanically demanding. The cartilage jaws of durophagous stingrays are known to be reinforced relative to non-durophagous relatives, with a thickened external cortex of mineralized blocks (tesserae), reinforcing struts inside the jaw (trabeculae), and pavement-like dentition. These strategies for skeletal strengthening against durophagy, however, are largely understood only from myliobatiform stingrays, although a hard prey diet has evolved multiple times in batoid fishes (rays, skates, guitarfishes). We perform a quantitative analysis of micro-CT data, describing jaw strengthening mechanisms in *Rhina ancylostoma* (Bowmouth Guitarfish) and *Rhynchobatus australiae* (White-spotted Wedgefish), durophagous members of the Rhinopristiformes, the sister taxon to Myliobatiformes. Both species possess trabeculae, more numerous and densely packed in *Rhina*, albeit simpler structurally than those in stingrays like *Aetobatus* and *Rhinoptera*. *Rhina* and *Rhynchobatus* exhibit impressively thickened jaw cortices, often involving >10 tesseral layers, most pronounced in regions where dentition is thickest, particularly in *Rhynchobatus*. Age series of both species illustrate that tesserae increase in size during growth, with enlarged and irregular tesserae associated with the jaws’ oral surface in larger (older) individuals of both species, perhaps a feature of ageing. Unlike the flattened teeth of durophagous myliobatiform stingrays, both rhinopristiform species have oddly undulating dentitions, comprised of pebble-like teeth interlocked to form compound “meta-teeth” (large spheroidal structures involving multiple teeth). This is particularly striking in *Rhina*, where the upper/lower occlusal surfaces are mirrored undulations, fitting together like rounded woodworking finger-joints. Trabeculae were previously thought to have arisen twice independently in Batoidea; our results show they are more widespread among batoid groups than previously appreciated, albeit apparently absent in the phylogenetically basal Rajiformes. Comparisons with several other durophagous and non-durophagous species illustrate that batoid skeletal reinforcement architectures are modular: trabeculae can be variously oriented and are dominant in some species (e.g. *Rhina*, *Aetobatus*), whereas cortical thickening is more significant in others (e.g. *Rhynchobatus*), or both reinforcing features can be lacking (e.g. *Raja*, *Urobatis*). We discuss interactions and implications of character states, framing a classification scheme for exploring cartilage structure evolution in the cartilaginous fishes.

## Introduction

**FIGURE 1:**
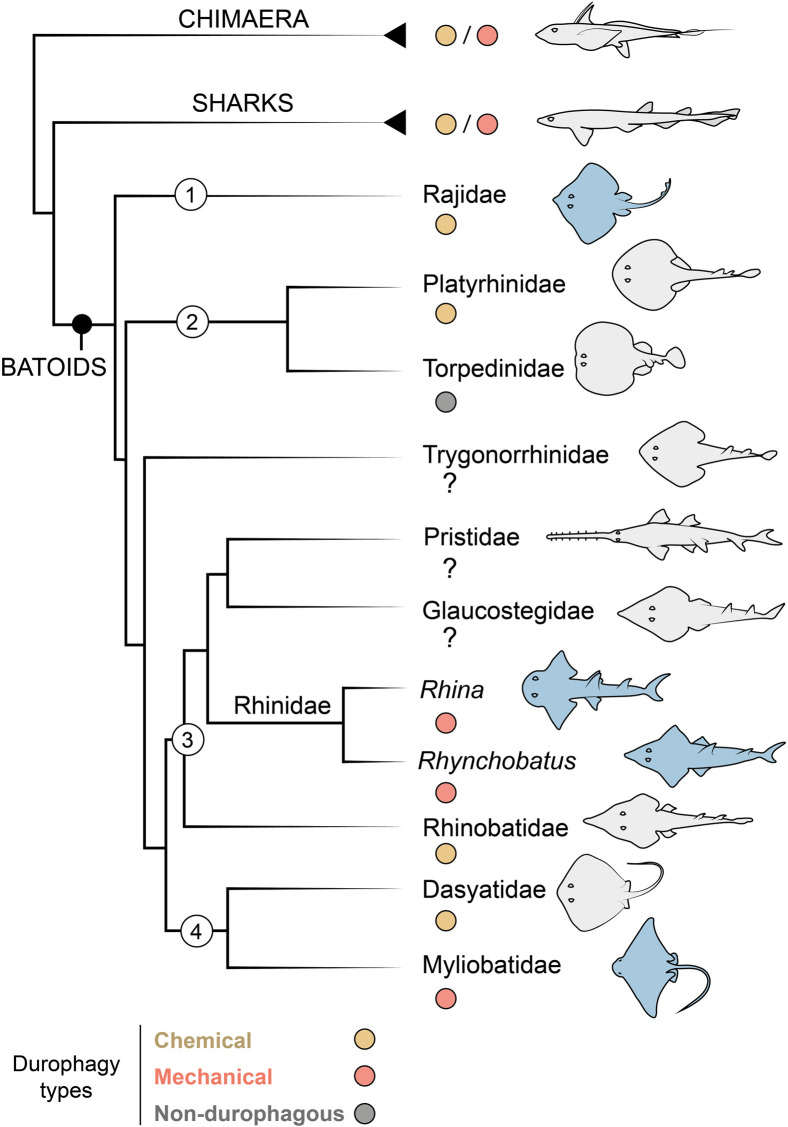
Durophagy in Chondrichthyes, with a focus on batoid relationships. Taxa of interest are indicated with numbers as follows: *1*) Rajiformes, *2*) Torpediniformes, *3*) Rhinopristiformes and *4*) Myliobatiformes. The Rhinidae is shown to genus level to indicate the two genera investigated in this study (*Rhina* and *Rhynchobatus*). Species examined in this study are highlighted in blue. In groups with some durophagous members, the most common types are highlighted, either chemical (where shelled prey is digested) or mechanical (where shelled prey is crushed). Groups where it is unclear if durophagy exists are marked with a question mark. Phylogenetic relationships based on (Franklin et al., 2014; Underwood et al., 2015).

Among cartilaginous fishes (Chondrichthyes), the consumption of hard prey (durophagy) is most common in the clade of skates and rays (Batoidea; Elasmobranchii), particularly in the subfamilies Rhinopterinae and Myliobatinae (both Myliobatiformes: Myliobatidae), which contain only durophagous taxa (Figure 1). Durophagy in batoid fishes takes a variety of forms: diets can involve comparatively thin-shelled crustaceans, thick-shelled molluscs and/or prey with softer, tougher exoskeletons (e.g. shrimp or even insects) (Wilga and Motta, 2000; Kolmann et al., 2015b, 2016). Hard prey processing has not been extensively surveyed in batoid fishes, but at least two strategies exist (Figure 1): what we will call “chemical durophagy,” where predators rely on low stomach pH or chinitase to break down prey exoskeletons (Fänge et al., 1979; Holmgren and Nilsson, 1999; Cortés et al., 2008; Anderson et al., 2010) and “mechanical durophagy,” where predators crush prey before ingestion (Summers, 2000; Summers et al., 2004; Kolmann et al., 2015b; Rutledge et al., 2019; Ajemian et al., 2021). The limited current knowledge of the phylogenetic distribution of these two strategies suggests they could be mutually exclusive in batoid fishes (Figure 1), perhaps also indicating that these prey processing modes demand a level of anatomical and physiological specialization, in gut physiology for chemical durophagy and in skeletal reinforcement for mechanical durophagy.

From an anatomical perspective, mechanical durophagy is a particularly impressive dietary mode for elasmobranch fishes, as shark and ray skeletons are composed predominantly of unmineralized cartilage covered by a mineralized crust of blocks called tesserae, typically arranged in a monolayer merely hundreds of microns thick (Maisey, 2013; Atake and Eames, 2021; Berio et al., 2021; Maisey et al., 2021). A variety of morphological features have been found to be associated with mechanical durophagy (e.g. Summers, 2000; Summers et al., 2004; Dean et al., 2007, 2015; Herbert and Motta, 2018; Rutledge et al., 2019; Seidel et al., 2021; Huie et al., 2022), particularly within durophagous myliobatiform stingrays (Myliobatiformes): flat pavement-like teeth; large adductor muscles; relatively shortened jaws with high leverage, fused at the midline symphysis; and structural reinforcements of the jaw tissues in the form of thickening of the mineralized cortex (the tesseral layer) and/or mineralized struts (trabeculae) coursing through the unmineralized cartilage. Some of these structural features, however, may support functions not associated with durophagy. For example, the cownose ray (*Rhinoptera bonasus*; Myliobatiformes) was previously considered to be an obligate durophage, its jaws bearing all the anatomical indicators of durophagy, yet this species has also been shown to suction feed opportunistically on soft-bodied prey (Collins et al., 2007). Conversely, the jaws of the non-durophagous electric ray, *Narcine brasilinensis* (Torpediniformes) have a thickened cortex and trabeculae, but these features likely support this species’ predation on buried polychaetes, preventing the highly protrusible jaws from buckling when they are used in benthic excavation of prey (Dean et al., 2006). The disparate feeding modes and phylogenetic positions of Myliobatiformes and Torpediniformes (Dean et al., 2006, 2007; Aschliman et al., 2012) suggest that reinforcing features such as trabeculae and cortical thickening may be more widespread in Batoidea than currently appreciated (Figure 1).

**FIGURE 2:**
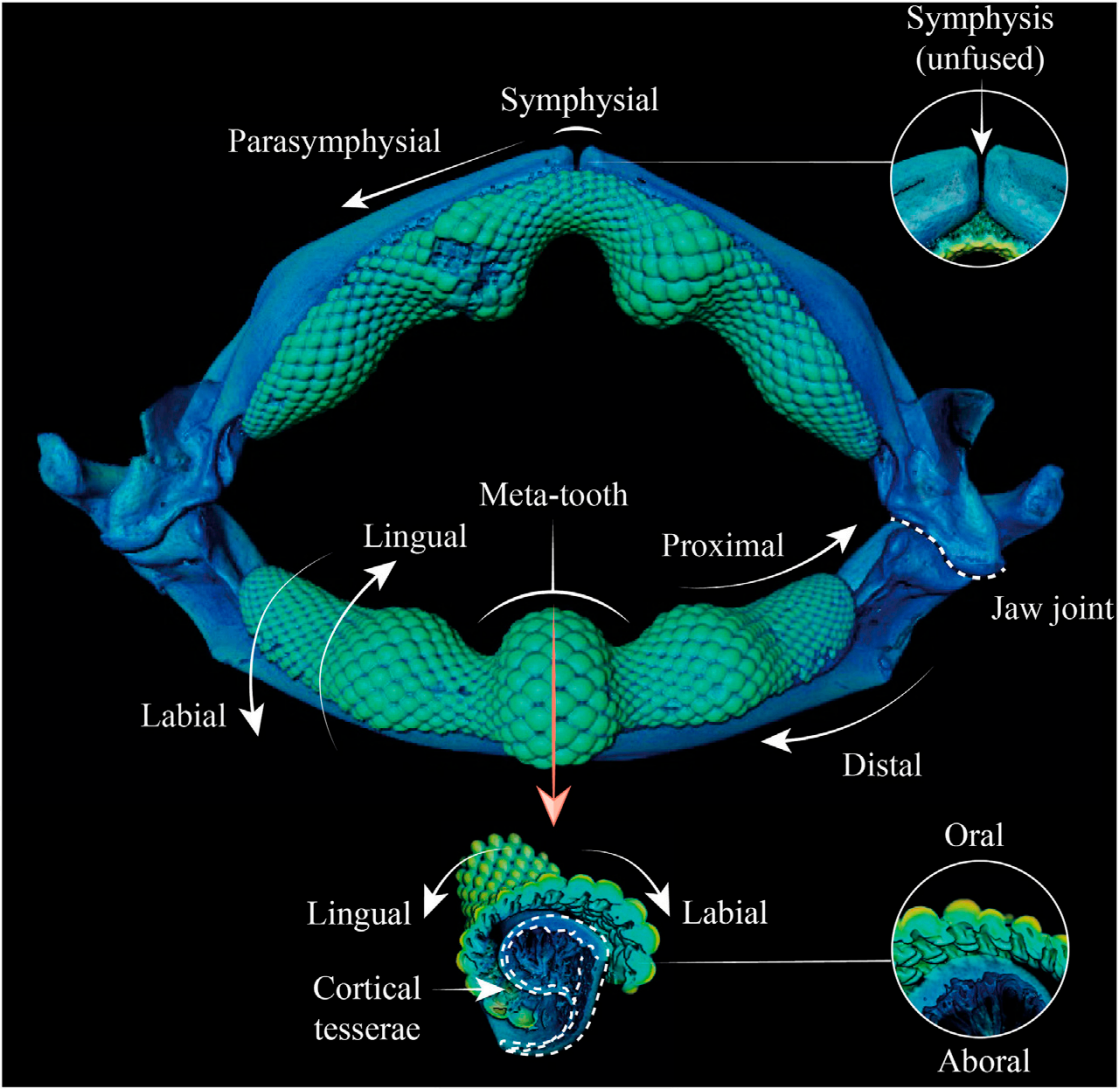
Anatomical terminology used in this study. 3D reconstruction (top) and cross-sections of the lower jaw (bottom) of *Rhina* indicate the different anatomical positions and orientation terminology used in the text.

**FIGURE 3:**
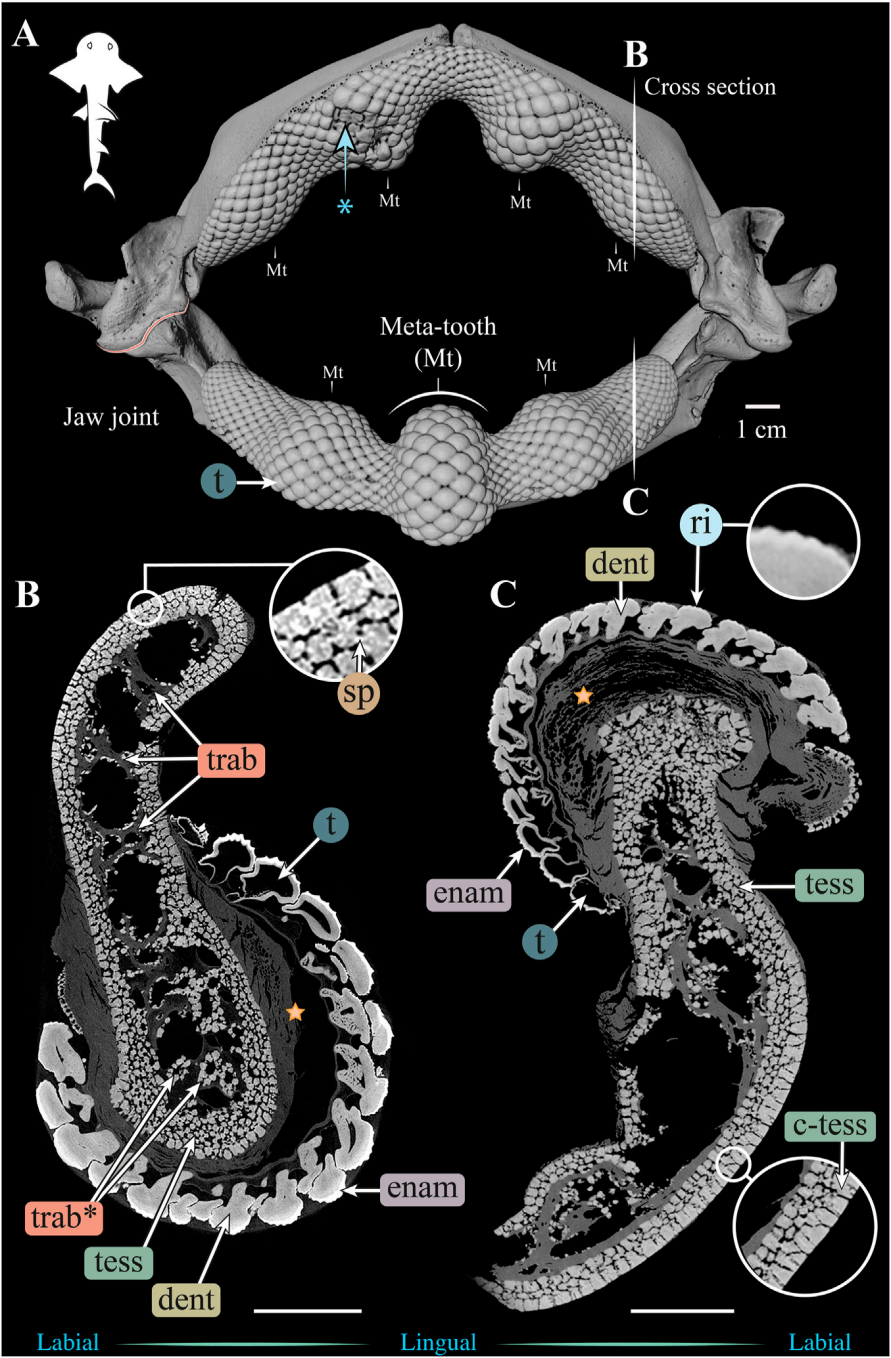
3D reconstruction and cross sections of a *Rhina ancylostoma* jaw. **(A)** 3D reconstruction of upper and lower jaws, showing the distinctive undulating dentition and several bulbous “meta-teeth” (Mt) on the upper and lower jaw. Asterisk indicates an area of the jaw with broken teeth. Vertical white lines indicate the position in the jaw of cross sections shown in **(B,C)**. Slices of the upper **(B)** and lower **(C)** jaws show trabeculae running parallel (trab) and perpendicular (trab*) to the section plane. Both globular (tess) and columnar (c-tess) tesserae form the jaw cortex, with hypermineralized “spokes” (sp) visible as regions of higher grayscale intensity, reinforcing the joints between tesserae. Tooth developmental stages can be distinguished in both sections, where newly formed teeth are hollow and progressively filled with mineralized dentin. Teeth typically exhibit surface ornamentation in the form of eight ridges, sculpted from the enameloid of each tooth crown. A bulk of unmineralized connective tissue (including jaw perichondrium and dental ligament) is visible between the teeth and tesserae (star). All scale bars 1 cm. Abbreviations: dent, dentin; enam, enameloid; ri, ridges; sp, spokes; tess, tesserae; c-tess, columnar tesserae; t, tooth; trab, trabeculae. Images from sample BMNH 2015.1.25.5 (24 cm).

**FIGURE 4:**
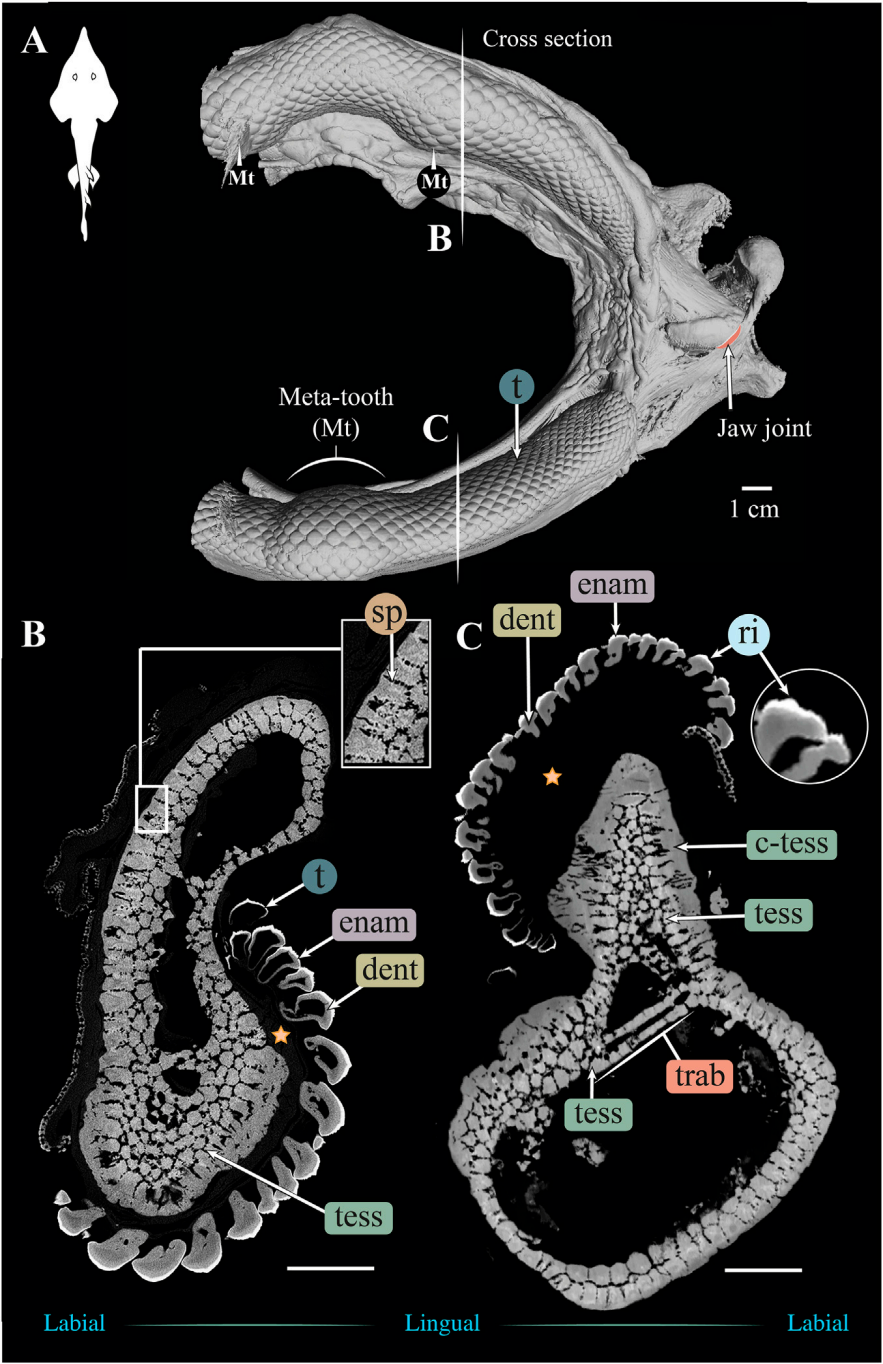
3D reconstruction and cross section of a *Rhynchobatus australiae* jaw. **(A)** 3D reconstruction of upper and lower jaws, showing the distinctive undulating dentition and several bulbous “meta-teeth” (Mt) on the upper and lower jaw. Vertical white lines indicate the position in the jaw of cross sections shown in **(B,C)**. Slices of the upper **(B)** and lower **(C)** jaws show trabeculae (trab) running largely parallel to the section plane (compare with the *Rhina* jaw in Figures 3B,C). Both globular (tess) and columnar (c-tess) tesserae form the jaw cortex, with hypermineralized “spokes” (sp) visible as regions of higher grayscale intensity, reinforcing the joints between tesserae (compare with the cortex of the *Rhina* jaw in Figure 3B, constructed from more numerous and smaller tesserae). Tooth developmental stages can be distinguished in both sections, where newly formed teeth are hollow and progressively filled with mineralized dentin. Teeth typically exhibit surface ornamentation in the form of low ridges, sculpted from the enameloid of each tooth crown. A bulk of unmineralized connective tissue (including jaw perichondrium and dental ligament) is visible between the teeth and tesserae (star). All scale bars 1 cm. Abbreviations: dent, dentin; enam, enameloid; ri, ridges; sp, spokes; tess, tesserae; c-tess, columnar tesserae; t, tooth; trab, trabeculae. Images from sample BMNH 2017.7.11.1 (28 cm).

In the last ∼20 years, much of the research into both elasmobranch skeletal biology and the functional morphology of durophagy has centered on myliobatiform stingrays (e.g. Summers, 2000; Summers et al., 2004; Dean et al., 2009; Kolmann et al., 2015a, 2015b; Seidel et al., 2016, 2017, 2021; Rutledge et al., 2019). Yet, batoid taxa offer a valuable diversity of species for exploring links between skeletal anatomy and ecology and clarifying how a cartilage skeleton can be modified through evolution to meet diverse functional demands. In this study, we use X-ray microcomputed tomography (micro-CT) to investigate whether jaw and dentition characters associated with stingray durophagy are also present in two durophagous members of the Rhinopristiformes, sister taxon to the Myliobatiformes (Dean et al., 2007; Aschliman et al., 2012; Aschliman, 2014) (Figure 1). We investigate two rhinopristiform species, *Rhina ancylostoma* (Bowmouth guitarfish; Rhinidae) and *Rhynchobatus australiae* (White-spotted wedgefish; Rhynchobatidae), large-bodied species with small, rounded, and ornamented teeth. In both species, these teeth form an unusual and striking dental battery, where multiple teeth are arrayed into spheroidal “meta-teeth”—bulbous structures constructed from multiple teeth, and particularly massive in *Rhina*—fitting into concave regions in the opposing jaw (Figures 2, 3A, 4A). These undulating dentitions are conspicuously different from the familiar, flat pavements of myliobatiform stingray teeth (e.g. Underwood et al., 2015), suggesting that the Rhinopristiformes may employ alternative anatomical strategies for durophagy.

Given that much previous research into cartilaginous fish durophagy has focused on myliobatiform stingrays, the Rhinopristiformes offer great potential for understanding the degree to which known anatomical modifications for a hard prey diet are group-specific or more general tissue-level modifications. Biomaterials and functional morphology studies (e.g. Summers, 2000; Dean et al., 2006; Liu et al., 2014; Seidel et al., 2019, 2020) have shown that tessellated cartilage (at least in some stingrays) has a distinct multi-scale mechanical anisotropy, with tesserae oriented parallel to the direction of loading (e.g. the biting direction) having a higher stiffness than those oriented perpendicular to it. Similarly, excised blocks of jaw cartilage are stiffer when they contain trabecular struts, and when the struts (and the tesserae forming them) are oriented in-line with the direction of applied load (Summers et al., 1998). The effect of tesserae orientation on skeletal stiffness presents a structural conundrum: the jaws of myliobatiform stingrays must have an appreciably broad, flat area to accommodate their pavement-like dentitions, yet this necessitates a wide skeletal surface where tesserae are oriented perpendicular to the biting load (i.e. in their less stiff orientation). This may explain the incredibly high density of trabeculae supporting the jaws in myliobatiform rays (i.e. buttressing occlusal areas with more tesserae oriented in-line with biting loads), while also suggesting that such supporting mechanisms may be less relevant for those durophagous batoids that lack flattened platform regions on their jaws (e.g. Rhinopristiformes) and/or that structural reinforcement may be accomplished by other means. It is possible, for example, that the shape of tesserae beneath the dentition may be altered, perhaps taking on the dome-like “voussoir” tesserae morphology known to be associated with arched skeletal surfaces (Maisey et al., 2021). Additionally, the jaw’s cortex could be reinforced by accessory tesseral layers (>10 have been described in some large species; Dean et al., 2015, 2017). We dissect these options in the current study, providing the first three-dimensional characterization of jaw-strengthening anatomies in batoids, comparing features among the range of durophagous and non-durophagous species examined in this study and previous works, to synopsize the diversity of strategies by which cartilage has been modified throughout elasmobranch evolution to meet varied performance demands.

## Materials and methods

### Sample selection and X-ray tomography acquisition

The dried jaw specimens examined in this study are from the Life Sciences Collections, Natural History Museum, London (BMNH) (Supplementary Table S1). Two specimens were used for the bulk of detailed analysis—*Rhina ancylostoma* (BMNH 2015.1.25.5) and *Rhynchobatus australiae* (BMNH 2017.7.11.1)—with additional higher-resolution scans focused on the regions of interest at the proximal ends of the lower and upper jaws. Other specimens of different sizes—*Rhina* (BMNH 2014.11.11.1) and *Rhynchobatus* (NHMUK PV P4048 and two unregistered specimens) — were scanned to investigate how tesserae and jaw trabeculae vary with age. The original body sizes of the animals from which specimens came were unknown and so jaw size (i.e. the outer jaw width at the jaw joints) was used as a proxy for age (i.e. larger jaws were assumed to come from larger and therefore older animals). A previous study (Dean et al., 2017) estimated *Rhynchobatus* jaw width to be ∼7–11% of total length and our measurements from two intact *Rhina* specimens (95.5 and 147 cm TL) and dried jaws from six specimens from animals of known total length suggest a similar ratio (∼11–15% of TL). Based on available size at maturity information for both species (Last et al., 2016; Purushottama et al., 2020), the jaw specimens used in our study (Supplementary Table S1) are likely all from mature individuals, an assertion supported by the high degree of mineralization of the skeleton (Seidel et al., 2016). It should be noted that two of the *Rhynchobatus* jaws could not be confidently identified to species; although available information indicates that all *Rhynchobatus* species include some amount of hard-shelled prey in their diet (e.g. Darracott, 1977; Moazzam and Osmany, 2020; Purushottama et al., 2020). Additionally, for comparative purposes, the upper and lower jaws of the durophagous stingray *Aetobatus* ex. gr. *narinari* (BMNH 2015.1.25.4; Myliobatiformes) and of the non-durophagous skate *Raja clavata* (BMNH 2015.1.25.2; Rajiformes) were scanned and examined.

Micro-CT imaging was performed at the Imaging and Analysis Centre, Natural History Museum, London, using a Nikon Metrology HMX ST 225 with a reflection target. The eight specimens listed above were scanned, as well as several selected regions imaged at smaller voxel sizes in both *Rhina* and *Rhynchobatus*, producing 12 separate data sets. X-ray source conditions ranged from 100 to 190 kV adjusted for field of view differences and sample density. A range of voxel sizes were achieved (26–121 µm), with smaller image pixel sizes utilized for quantifying tesserae dimensions. For a detailed list of micro-CT scan parameters and specimen information, *see* Supplementary Information, Supplementary Table S1.

### Image processing

All scans were processed, rendered, and analysed using Avizo/Amira (version 9.4 and above or Amira ZIB Edition). Each acquisition was enhanced using a “low level” non-local means filter to reduce imaging noise from the data. Individual jaw elements were separated (segmented) for comparative analysis, using volume editing tools to isolate the lower jaw (Meckel’s cartilage) from the upper jaw (palatoquadrate), and to separate the left and right jaw moieties at the symphyses.

### Terminology

Anatomical terminology used is presented visually in Figure 2. “Proximal” indicates a position or direction toward the jaw joint and “distal” toward the jaw symphysis. “Symphyseal” refers to the (distal) midline joint between the two jaw halves, with “parasymphyseal” regions flanking the jaw symphysis laterally (e.g. Underwood et al., 2015). “Oral” is towards the biting surface of the jaw or teeth, with “aboral” indicating the opposing surface. “Labial” refers to the outer surface of the jaw, and “lingual” to the inner (pharyngeal) surface of the jaw. “Cortical” refers to a position or direction toward the jaw’s cortex, the outer mineralized rind comprising single or multiple layers of tesserae. “Perichondral” is used similarly, to indicate tesserae or portions of tesserae associated with the unmineralized, collagenous perichondrium that wraps the outer surface of tessellated cartilage skeletons (Seidel et al., 2020, 2021).

### Measurements

#### Cortical and tooth thickness

**FIGURE 5:**
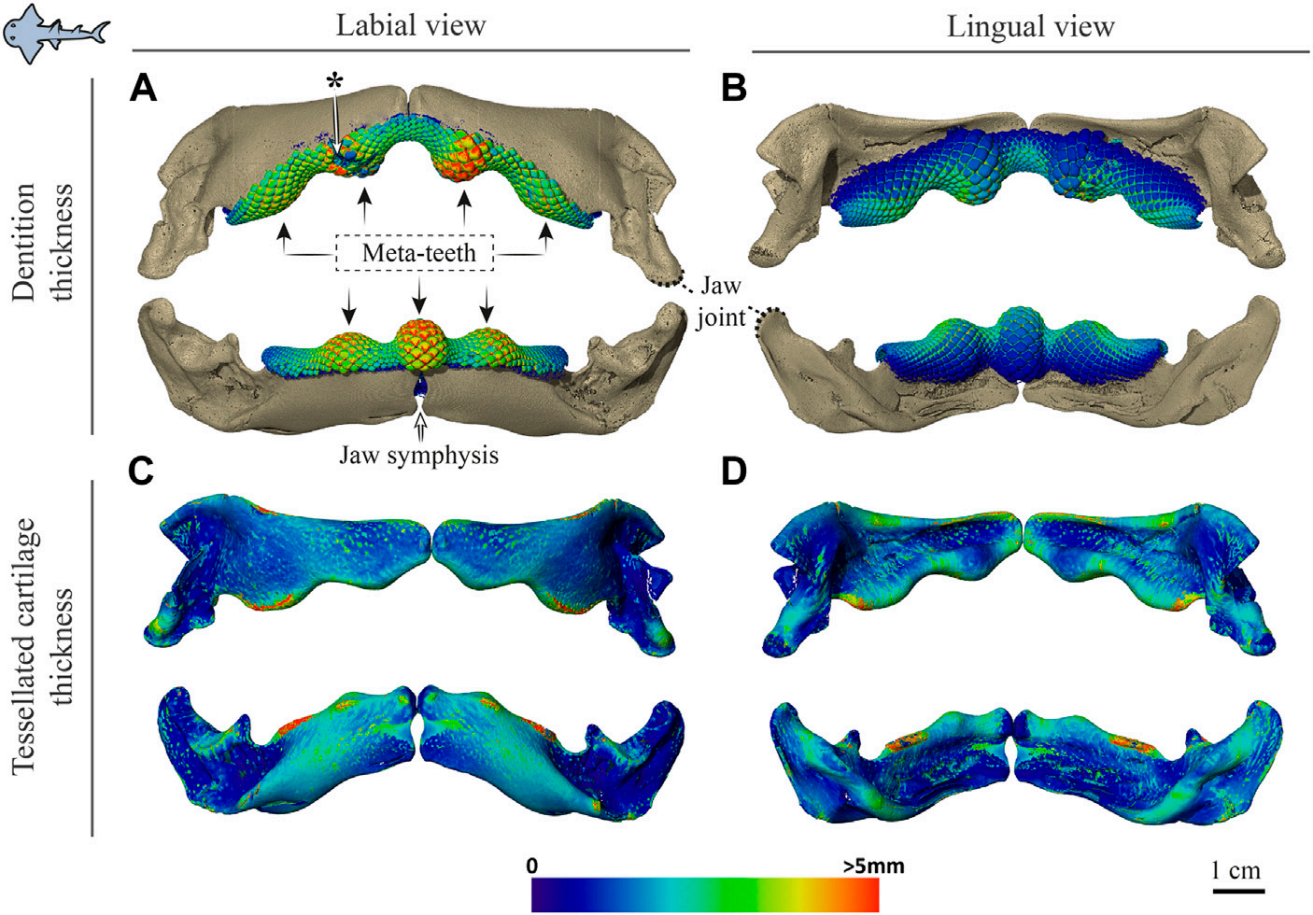
Dentition and tessellated cartilage thickness in the jaws of *Rhina ancylostoma.* **(A**,**B)** Color-coded dentition thickness, and **(C**,**D)** jaw cortex (tessellated cartilage) thickness. Both upper and lower jaws are shown in labial **(A**,**C)** and lingual **(B**,**D)** views. Black arrows in **(A)** indicate the positions of the meta-teeth in the lower and upper jaw. Asterisk in **(A)** indicates a region of tooth breakage (*see* also Figure 3A). Note also the gap between left and right jaw moieties, illustrating the lack of symphyseal fusion. Thickness is represented by a physics color map, with regions in red being thicker than 5 mm. Images from sample BMNH 2015.1.25 (24 cm).

**FIGURE 6:**
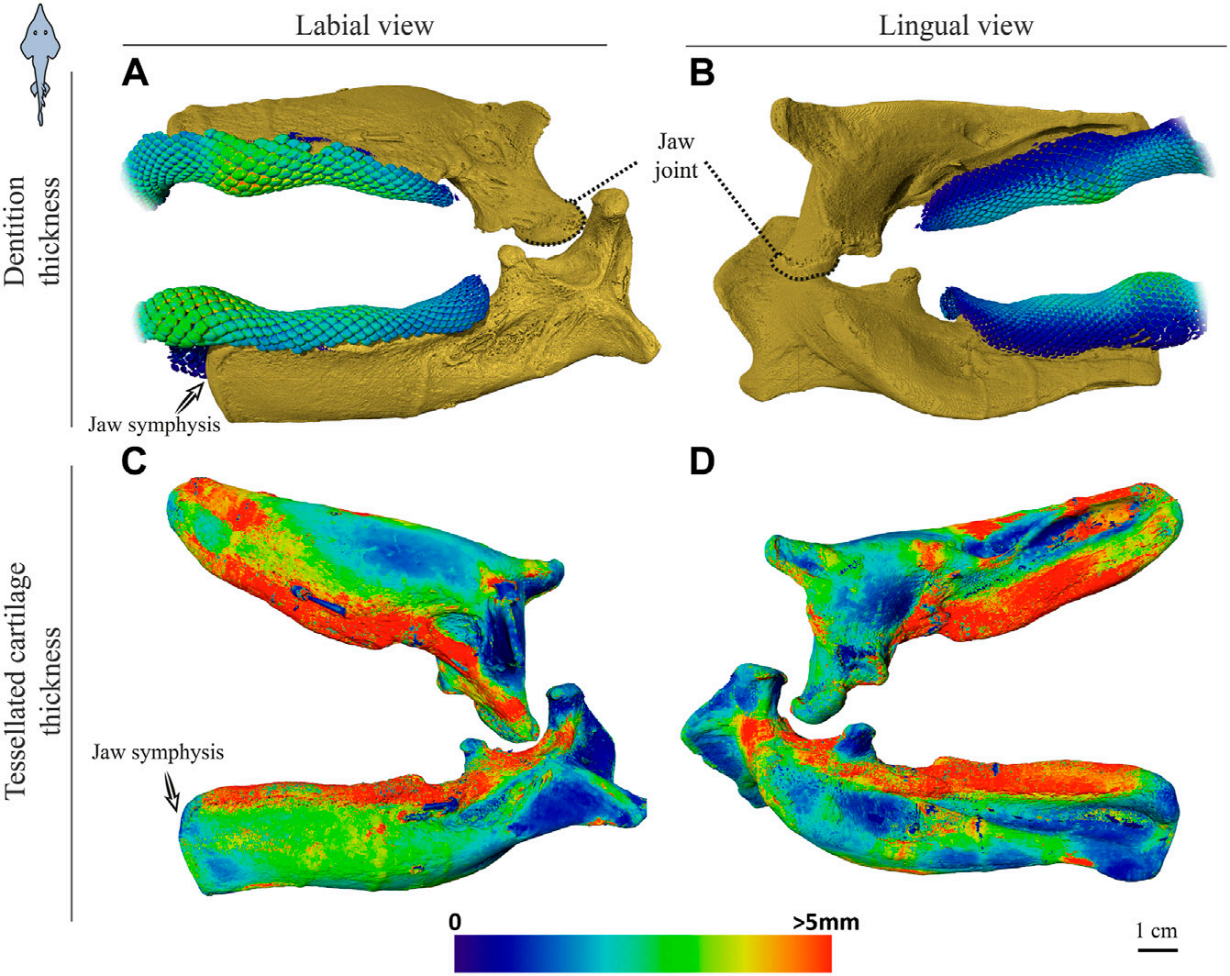
Dentition and tessellated cartilage thickness of the left jaw moieties of *Rhynchobatus australiae.* **(A**,**B)** Color-coded dentition thickness, and **(C**,**D)** jaw cortex (tessellated cartilage) thickness. Both upper and lower jaws are shown in labial **(A**,**C)** and lingual **(B**,**D)** views. As in the *Rhina* jaws (Figures 5C,D), the symphysis is unfused (note the anatomical edge of the jaw halves). Thickness is represented by a physics color map, with regions in red being thicker than 5 mm. Images from sample BMNH 2017.7.11.1 (28 cm).

**FIGURE 7:**
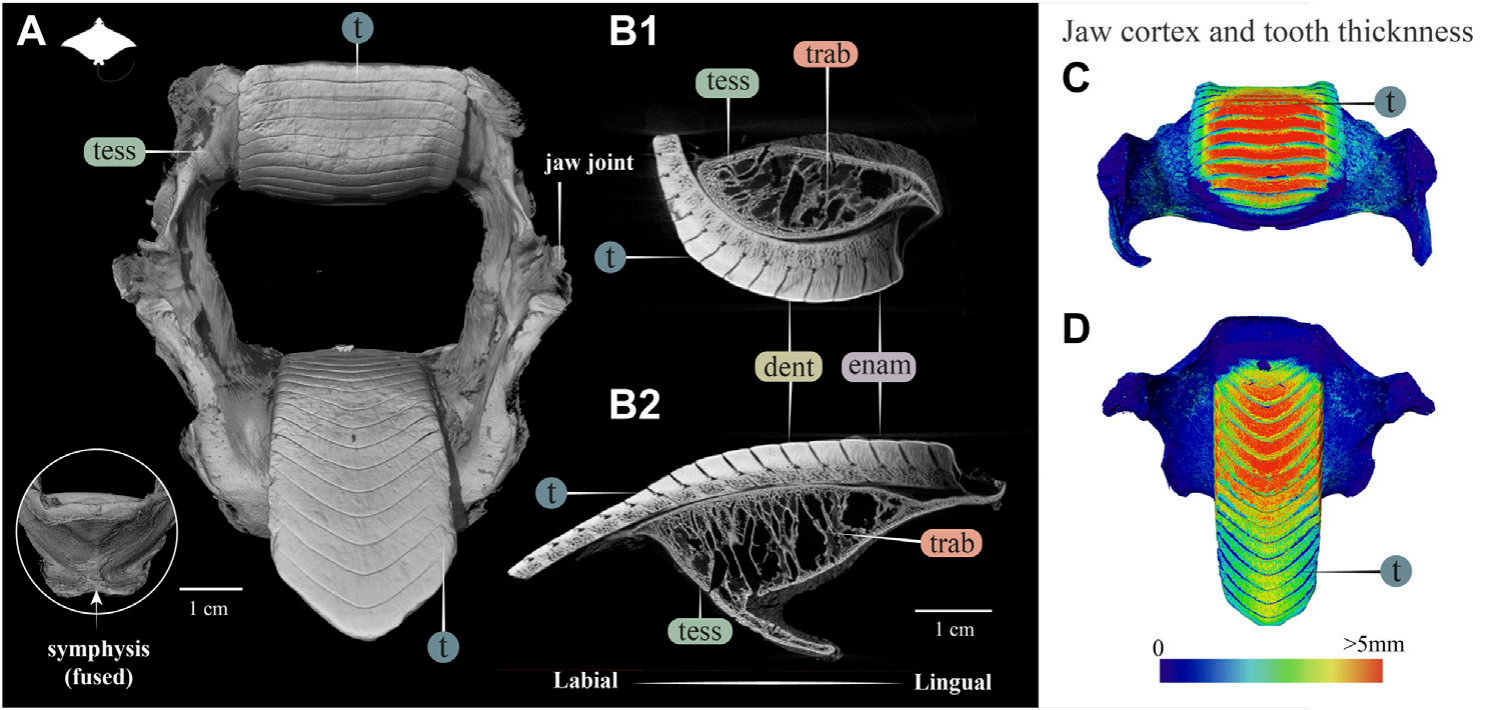
3D reconstruction, cross section and thickness analysis of the jaw of an eagle ray jaw (*Aetobatus* ex. gr. *narinari*). **(A)** 3D reconstruction of upper and lower jaw and teeth in labial view. The inset shows the fused symphysis beneath the dentition. **(B)** Slices through the opposing upper **(B1)** and lower **(B2)** jaws, showing a cross section of the jaw skeleton, comprising a cortex of tesserae and internal trabeculae, and surmounted by interlocked teeth. Note that the cortex is comprised of fewer layers of tesserae and trabeculae exhibit more of a hierarchical branching pattern than in the jaws of *Rhina* (Figure 3) and *Rhynchobatus* (Figure 4). A visible progression of teeth development can be seen (moving from right to left in B1 and B2), characterized by a more porous dentin in newly formed teeth, infilled with mineralized dentin as teeth become functional. **(C**,**D)** Jaw cortex and tooth thickness are represented by a physics color map with regions in red being thicker than 5 mm. Abbreviations: enam, enameloid; dent, dentin; tess, tesserae; t, tooth; trab, trabeculae. Images from sample BMNH 2015.25.4 (7.5 cm).

**FIGURE 8:**
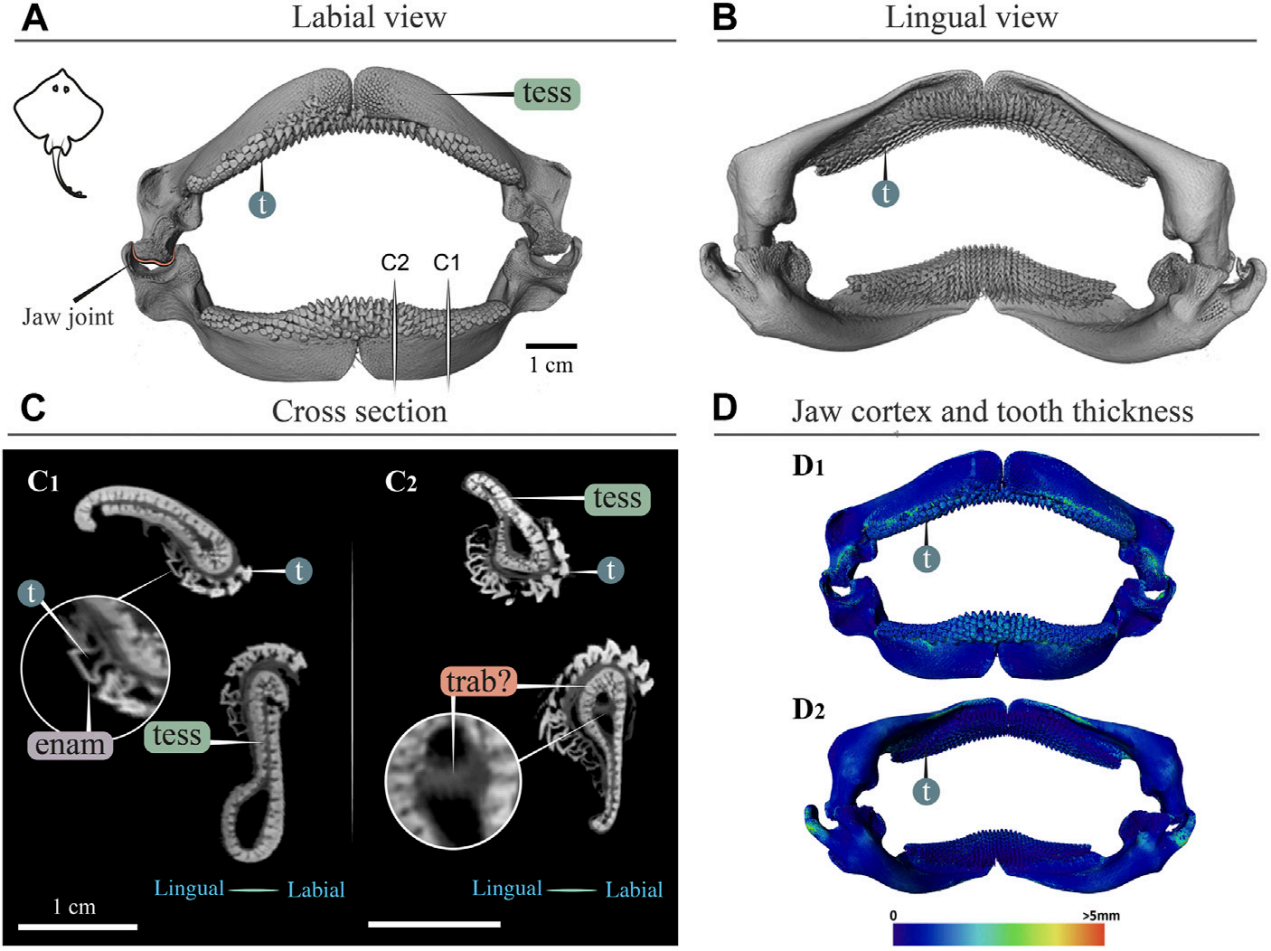
3D reconstruction, cross section and thickness analysis of a skate jaw (*Raja clavata*). 3D reconstruction of upper and lower jaw with labial **(A)** and lingual **(B)** views of the jaws and teeth. Vertical white lines in A indicate the position in the jaw of cross sections shown in C1 and C2. **(C)** Slices show cross sections of the upper and lower jaws at two positions **(C1**,**C2)**. Tooth developmental stages can be distinguished in both sections, where newly formed teeth are hollow and progressively filled with mineralized dentin. Note that the cortex comprises only a single layer of tesserae, with a single potential trabecula (trab?) passing through the jaw. **(D)** Jaw cortex and tooth thickness are represented by a physics color map with regions in red being thicker than 5 mm (**D1** labial view, **D2** lingual view). All scale bars 1 cm. Abbreviations: enam, enameloid; dent, dentin; tess, tesserae; t, tooth; trab?, potential trabecula. Images from sample BMNH 2015.25.2 (9 cm).

The thickness of the jaw cortex and the dentition was measured from the full jaws of *Rhina* (Figure 3) and *Rhynchobatus* (Figure 4), on a mesh generated from the segmented volumes of the whole jaw specimen scans. Meshes were generated for the upper and lower jaws, and the upper and lower dentitions for each species; the upper and lower jaw cartilages were analysed independently from the dentition. The use of meshes simplified the process of bulk linear measurements and allowed thickness to be color-coded over the entire jaw surface: meshed surfaces were minimally smoothed and simplified to reduce computational resources needed, then thickness was determined by measuring the distance between two opposing vertices in the mesh that were both nearest and parallel (or very close to parallel) to each other. To visualize thickness variation, measurements were then represented by a surface scalar field for each vertex and a physics color map with a range of 0 to >5 mm, with thickness above 5 mm represented as red (Figures 5, 6). This process allowed quantification and visualization of the bulk thickness of the jaw cortex (regardless of the number of tesseral layers) and the dentition (i.e. tooth height, including the contributions of enameloid and dentin). For comparative purposes, additional meshes were generated and similarly quantified for the upper and lower jaw cartilages and dentitions of *Aetobatus* ex. gr. *narinari* (Figure 7) and *Raja clavata* (Figure 8).

#### Tesserae

**FIGURE 9:**
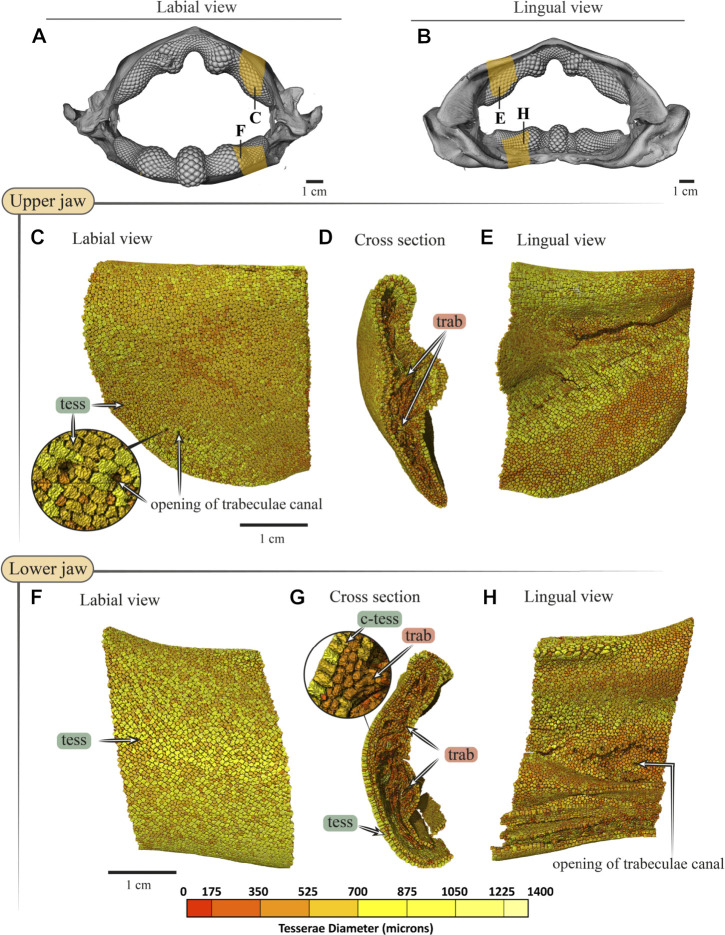
Tesserae size variation in the jaws of *Rhina ancylostoma.* **(A**,**B)** 3D rendering of *Rhina ancylostoma* jaws, in labial **(A)** and lingual **(B)** views, showing the regions of interest (yellow) used for tesserae size analysis. The scale for tesserae size color-coding is shown at the bottom of figure. **(C**–**E)** Tesserae color-coded according to their size in labial, cross-section and lingual views of the upper **(C**–**E)** and lower **(F**–**H)** jaw. The pores visible in the labial cortical surface are openings for trabeculae canals **(C**,**H)**, and volume-rendered trabeculae can be seen running through the cross-section **(D**,**G). (F**–**H)** Columnar tesserae (c-tess) form the superficial portion of the cortex in both upper and lower jaws. Note that tessera size is not uniform, but rather varies across the cortical surface. All scale bars 1 cm. Abbreviations: c-tess, Columnar tesserae; tess, tesserae; trab, trabeculae. Full jaw images are from sample BMNH 2015.1.25.5 (24 cm) and regions of interest images are from sample BMNH 2014.11.11.1 (35 cm).

**FIGURE 10:**
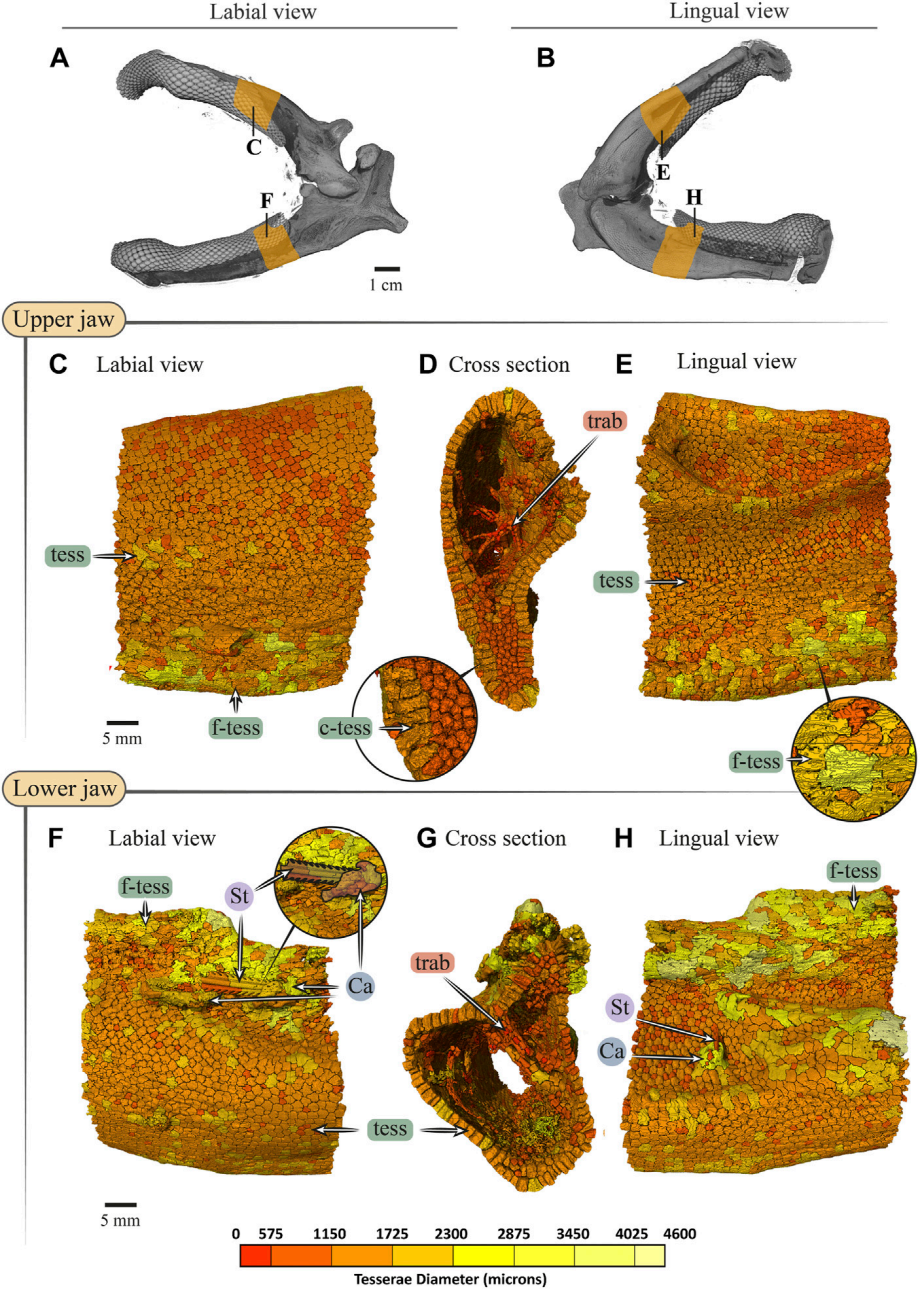
Tesserae size variation in the jaws of *Rhynchobatus australiae.* **(A**,**B)** 3D rendering of *Rhynchobatus australia*e jaws, in labial **(A)** and lingual **(B)** views, showing the regions of interest (yellow) used for tesserae size analysis. Tesserae color-coded according to their size in labial, cross section and lingual views of the upper **(C**–**E)** and lower **(F**–**H)** jaw. Volume-rendered trabeculae can be observed running across the cross sections of the upper **(D)** and lower jaws **(G)**. Columnar tesserae (c-tess) form the superficial portion of the cortex **(inset in D**,**G)**. In labial **(F)** and lingual **(H)** views, a barb/sting from a stingray can be observed embedded in the jaw, being surrounded with a mineralized callus (*see* Dean et al., 2017). Note that tessera size is not uniform, but rather varies across the cortical surface. Abbreviations: Ca, callus; St, sting; tess, tesserae; c-tess, columnar tesserae; f-tess, fused tesserae; trab, trabeculae. Full jaw images are from sample *Unregistered specimen* (11 cm) and region of interest Images are from sample BMNH 2017.7.11.1 (28 cm).

**FIGURE 11:**
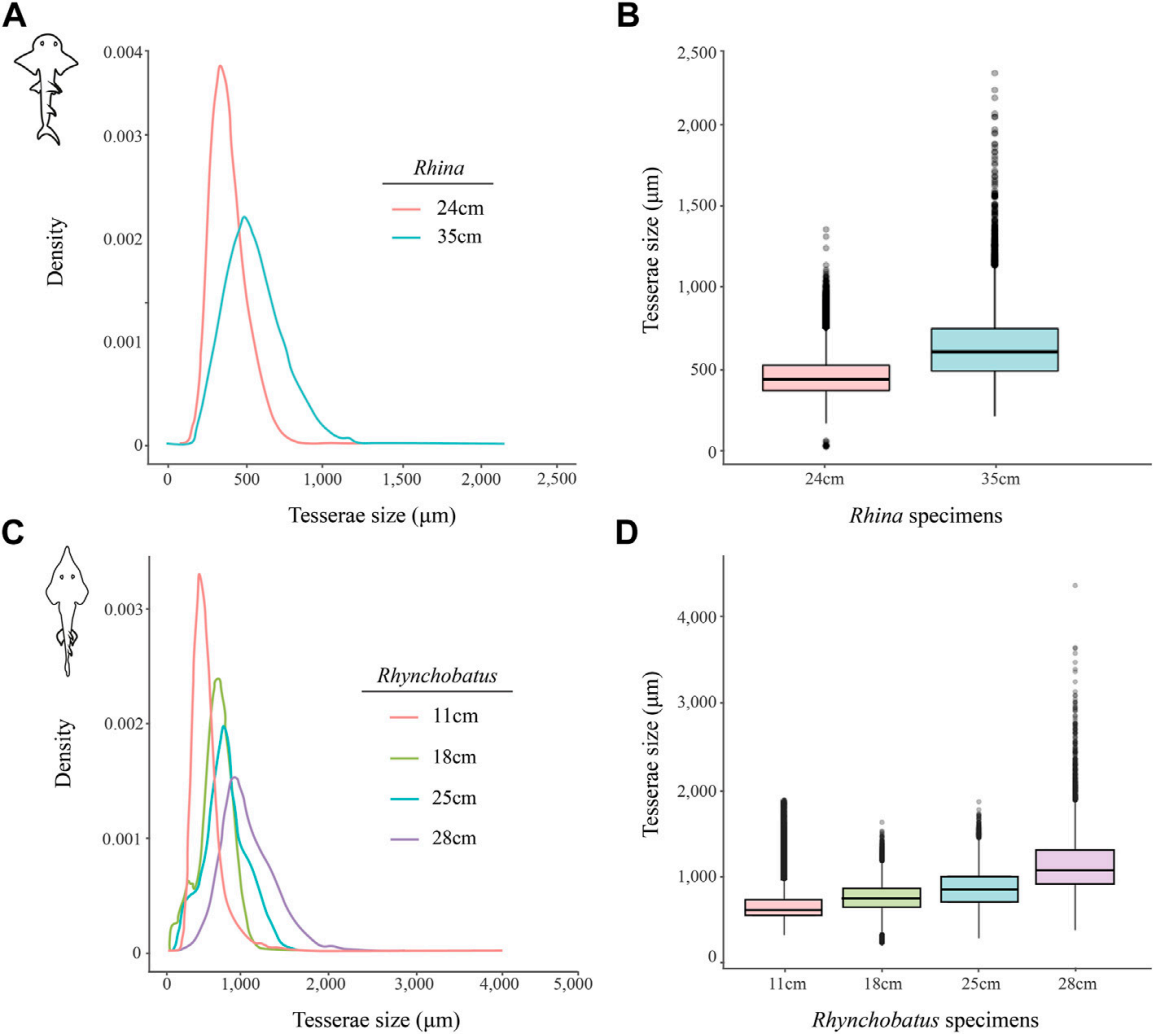
Variation of tesserae size across different jaw sizes (ages) in *Rhina* and *Rhynchobatus.* **(A**,**B)** Density graph **(A)** and boxplot **(B)** showing the distribution differences of tesserae size between two different *Rhina* specimens of different jaw sizes. **(C**,**D)** Density graph **(C)** and boxplot **(D)** showing the distribution differences of tesserae size among four *Rhynchobatus* specimens of different jaw sizes. All graphs illustrate an increase in tesseral size and a broadening of the size distributions with age. Note the size scale differences between the *Rhina* and *Rhynchobatus* graphs, with relatively larger tesserae in *Rhynchobatus*.

Qualitative evaluation of the arrangements and morphologies of tesserae in the jaw cortex were performed on scan slice data and volume-renderings. To quantify aspects of tesseral morphology, the smallest possible voxel size was necessary to resolve tesserae boundaries successfully, but this came at the expense of scan volume: whereas entire jaws could be scanned from smaller specimens (i.e. the two smallest *Rhynchobatus* jaws, 11 and 18 cm [unregistered specimens]), ROI-scans were necessary to quantify tesserae morphometrics in larger specimens (lower right jaws of *Rhynchobatus*, NHMUK PV P4048 and BMNH 2015.1.25.5; lower right jaw of *Rhina*, BMNH 2014.11.11.1 and 2015.1.25.5; Supplementary Table S1). The dimensions of individual tesserae were investigated in the entire jaws of the small specimens and across four ROIs at the proximal ends of both the upper and lower jaws of the larger *Rhina* and *Rhynchobatus* (Figures 9, 10 and Supplementary Table S2); these regions were chosen for their comparatively simple cross-sections and the fact that they could be consistently compared across individuals and species (avoiding the undulating morphologies of the symphyseal regions, which become more pronounced with age). The mineralized tissue was segmented and individual tesserae isolated using the Separate Objects module with a marker extent of “2” (relating to the size of the seeds marking objects for separation). Segmentation of individual tesserae was possible due to the narrow gaps between tesserae (i.e. intertesseral joints) and with a high level of accuracy, particularly where high magnification was achieved. The Separate Objects module applies the Chamfer method which splits volumetric bodies that touch only partially with neighbouring bodies (e.g. at the “intertesseral contact zones,” where tesserae abut; Seidel et al., 2016; Jayasankar et al., 2020). Using this method, structures with strong overlap are not separated. In our data, once tesserae were segmented from one another, we recorded their locations (X, Y and Z coordinates of their centroids) and also approximated their sizes by measuring the major, intermediate and minor axis lengths of a bounding box enclosing all of the voxels belonging to each tessera (Supplementary Figure S1). Although this high-throughput size estimation calculates the length, width and thickness of every tessera’s bounding box, it does not determine how the bounding box’s orientation is linked to the tessera’s anatomical orientation. In other words, the method can calculate, for example, the longest dimension of a tessera’s bounding box, but not whether this is the tessera’s “width” or its “thickness” (i.e. the dimension parallel to the surface of the jaw vs perpendicular to it; *see* Supplementary Figure S1); addressing this challenge requires a method for identifying which tesseral face is associated with the skeletal surface, a feature unavailable in the segmentation and analysis software. As a result, in the current study, we only report the bounding box’s smallest linear dimension as an indication of “tesseral size.” We found this to be a reliable and usefully conservative general measurement for illustrating local variations in tesseral size, particularly since the longest bounding box dimension is heavily biased by tesseral fusions (*see* Results) and since length is a more anatomically intuitive metric than volume for tesserae. Measured values for tesserae size (Supplementary Table S2) were in reasonable agreement with 2D measurements from previous works (e.g. Dean et al., 2009; Seidel et al., 2016, 2021; Maisey et al., 2021), supporting the validity of our method. Comparisons of the various possible tesseral measurements and the development of methods for anatomical orientation of bounding boxes will be addressed in a future work (B. Yang et al., in preparation). Tesserae size distributions were plotted for all specimens of both species across eight size bins, distributed equally between each species’ minimum and maximum tesseral sizes (Figure 11). Tesseral size was also represented in jaw volume renderings with color maps grading from dark red to pale yellow (Figures 9, 10 and Supplementary Figures S1, S2, Supplementary Table S2).

#### Trabeculae

**FIGURE 12:**
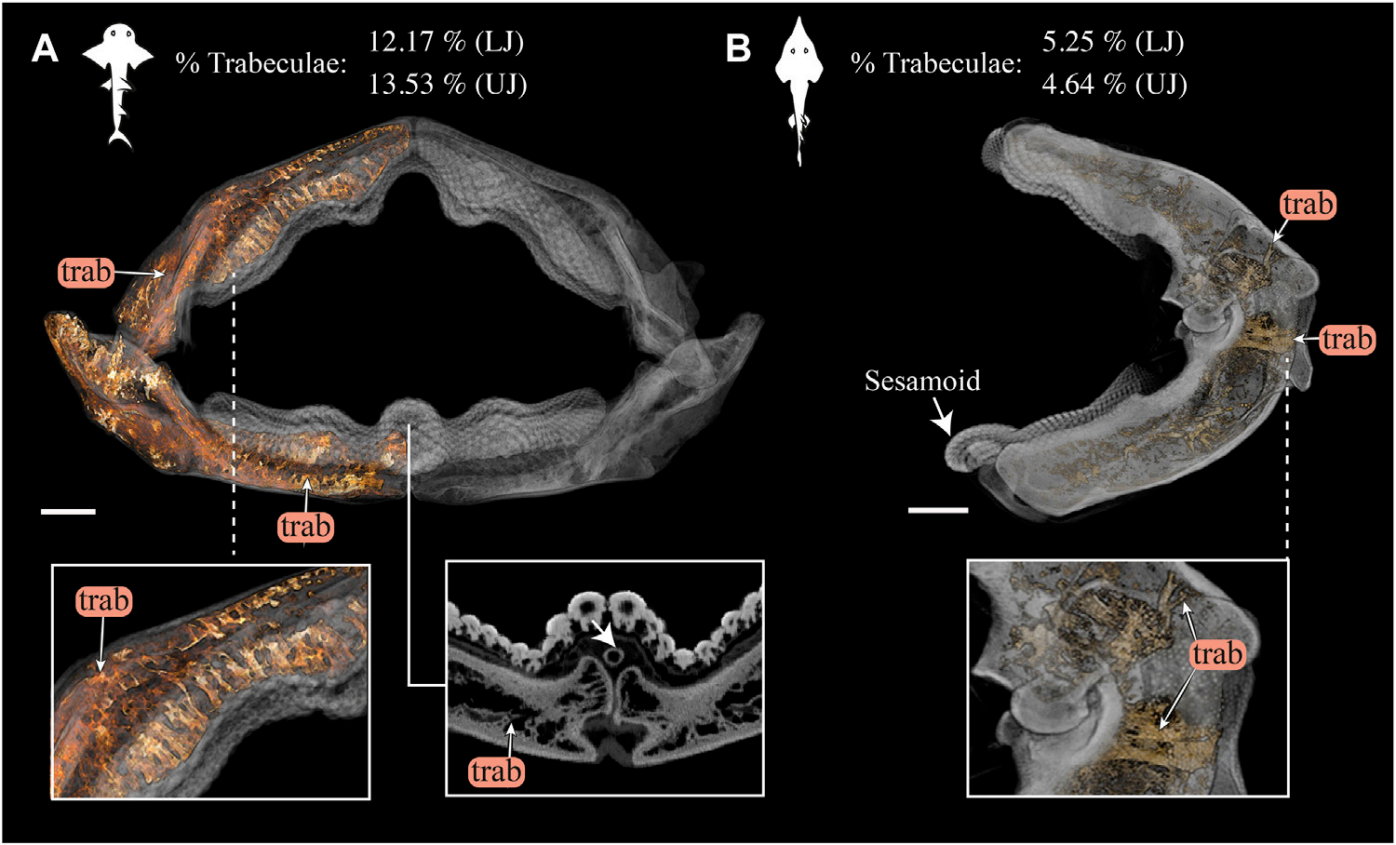
3D rendering and volumetric measures of trabeculae in *Rhina* and *Rhynchobatus* jaws. **(A)** 3D rendering of the full jaw of *Rhina ancylostoma*, with trabeculae (trab) highlighted in orange. A higher magnification view (left inset) shows trabeculae of relatively consistent orientation in the parasymphyseal region and a cross-section of the meta-teeth (right inset) shows the sesamoid cartilage (white arrow). **(B)** 3D rendering of the left jaw moiety of *Rhynchobatus australiae*, showing that, although less dense than in *Rhina*, trabeculae are also present*,* especially in the region surrounding the jaw joint (bottom inset). The mandibular sesamoid of *Rhynchobatus* is visible in the volume rendering in **(B)** (white arrow). The interspecies difference in trabecular density is also quantified in terms of volume percentage of trabeculae at the top of the figure. All scale bars 1 cm. Abbreviations: trab, trabeculae. Images from samples BMNH 2015.1.25.5 (*Rhina ancylostoma,* 24 cm) and BMNH 2017.7.11.1 (*Rhynchobatus australiae,* 28 cm).

Trabeculae position and orientation were examined from scan slice data and volume-renderings. In addition, trabeculae were manually segmented from the full jaw scans of both species—the right upper and lower jaw moieties in *Rhina* (BMNH 2015.1.25.5) and the left upper and lower jaw moieties in *Rhynchobatus* (BMNH 2017.7.11.1) — by combining region-growing and selective thresholding, using a brush tool to separate the trabeculae from the cortical mineralized cartilage. Trabeculae prevalence was then quantified as a percent volume fraction relative to the total volume of mineralized jaw cartilage (including trabeculae; Supplementary Table S3). To further visualize interspecies differences, trabeculae were also volume-rendered using a red to light yellow color map with the remaining (non-trabecular) mineralized tissue (i.e. the jaw cortex) visualised in transparent grayscale (Figure 12).

## Results

### *Rhina* and *Rhynchobatus*

#### Gross morphology, jaw cortex and cortical tesserae

Although similar-sized jaws were examined for *Rhina* and *Rhynchobatus*, jaw morphologies and tissue arrangements varied considerably (Figures 3, 4). Our scans revealed that the oral/occlusal edges of both species’ jaws echo the undulating morphology of their meta-tooth arrays. The symphyseal regions were exceptions to this, where the largest meta-teeth (on the lower jaw in both species) encircled the unfused symphyseal joint (labeled meta-tooth in Figures 2–4). The symphyseal mandibular meta-tooth was most striking in *Rhina*, forming a robust, denticulate torus around the symphysis (Figure 3). The meta-teeth themselves were not solid masses of mineralized tissue, but rather spheroidal constructions of slightly larger-than-average teeth (*see* section 3.1.3 below), forming a shell around an underlying mass of unmineralized dental ligament and perichondrium (star in Figures 3B,C, 4B,C). In both species, a single ovoid tessellated sesamoid cartilage was seen embedded in the connective tissue of the mandibular meta-tooth, between the tooth layer and symphysis (Figure 12 and Supplementary Figure S1).

Significant differences were also apparent between the species in the thickness of the jaw cortex, how this thickness was constructed, and in the morphologies of the tesserae themselves. The general gross tissue arrangements, however, were similar between the species: beneath the teeth, the jaws were roughly sigmoid or pear-shaped in cross-section, with a pronounced lingual depression housing the youngest portion of the tooth array (hollow, incipient teeth; *see* section 3.1.3 below) and with connective tissue linkages between the tooth array and jaw cortex as described above (dark areas marked with stars in Figures 3B,C, 4B,C). The thickness of the mineralized cortex (composed of tesserae) varied across regions of the jaws in both taxa, in general thickest along the oral jaw surfaces, and thinnest near the jaw joint and symphysis (Figures 3B,C, 4B,C, 5C,D, 6C,D). In *Rhina*, the cortex was generally between 0.5 and 1.5 mm thick, however with the cortex visibly thicker along the biting (oral) and contralateral aboral margins (∼2–3 mm and up to nearly 5 mm in some areas) (Figures 5C,D). The labial side of the lower jaw was also noticeably thicker than the contralateral lingual face (∼2–3 mm; Figures 5C,D), this thickened cortex continuing from the symphysis to the area of the proximal termination of the dentition, approaching the jaw joint. In *Rhynchobatus*, the cortex of the upper and lower jaws was overall notably thicker than in *Rhina* (in most areas >3 mm), with the labial cortex often more than 1 mm thicker than the lingual, and the oral jaw margins strikingly reinforced (>5 mm thick; Figures 6C,D).

In both *Rhina* (Figures 3B,C, Supplementary Figures S2B,C, Supplementary Movies S1, S2) and *Rhynchobatus* (Figures 4B,C, Supplementary Figures S1B,C, Supplementary Movie S3), the jaw cortex is formed from multiple monolayers of tesserae. In virtual sections, a high variation in tesseral shape and size is apparent, with tesserae noticeably larger and fewer in number in *Rhynchobatus* than in *Rhina*, even for jaws of similar sizes (Figures 3B,C versus Figures 4B,C). Unlike the thin plate-like tesserae described for other batoid species (e.g. *see* Fig. 10 in Seidel et al., 2016), in *Rhina* and *Rhynchobatus*, tesserae shapes tended toward columnar (tall and narrow; *c-tess* in Figures 3C, 4C, 9G, 10D) or globular forms (more common; Figures 3B,C, 4B,C), but varied locally across the cross-sections of both species’ jaws. In *Rhina*, tesserae in the labial jaw cortex tended to be larger and columnar in shape (Figures 3B,C), in comparison with tesserae of the lingual cortex, which were smaller and arranged in fewer layers, particularly in the lower jaw (Figure 3C). In *Rhynchobatus*, labial and lingual tesserae were more comparable in size (Figures 4B,C).

Within the multiple tesseral layers of the jaw cortex, the outermost/surface layer typically had a more regular morphology (in size and shape) relative to the inner layers, readily distinguishable by eye (*see* below; e.g. Figures 3B,C, 4B,C). Most jaw regions examined in both *Rhina* and *Rhynchobatus* exhibited from three to seven layers of tesserae, with the most numerous tesseral layers observed in association with the jaw’s oral surface (beneath the teeth; Figures 3B,C, 4B,C). Oral (sub-dental) multi-layers were observed even in the smallest specimen examined (*Rhynchobatus*, Supplementary Figures 1B,C), although in the largest *Rhina*, the number of oral tesseral layers appeared reduced, compared to the medium *Rhina* (compare Figures 3B,C to Supplementary Figures 2B,C). In sub-dental regions, the number of tesseral layers was challenging to count, as the smaller tesserae there formed a disorganized scree nearly filling the narrow, labiolingually-compressed interior of the jaw (Figures 3C, 4C, 9D,G, 10D,G). This “tesseral scree” was also occasionally visible in the narrow aboral regions of the jaw (e.g. Figure 4B). Although a multi-layered tesseral cortex was the norm in both taxa, monolayers were present in localized areas of the aboral-lingual margin of the lower jaw of *Rhynchobatus* (Figure 4C). Convex regions of the jaw cortex in all scans had abundant columnar tesserae, often twice as thick as tesserae in flatter areas of the skeleton (Figures 3C, 4C, 9D,G, 10D,G). These tesserae were often somewhat wedge-shaped, slightly wider at their perichondral side.

The tesserae of both species were typically hundreds of micrometers wide, with those of *Rhynchobatus* on average larger than those of *Rhina* (note the scale differences in the color-coding in Figures 9–11 and Supplementary Table S2). Although only region-of-interest scans were performed for larger specimens, all datasets examined included tens of thousands of tesserae (and more than 100,000 tesserae in one case), with the small age series examined exhibiting the same trend for both species: with increasing jaw (and, therefore, animal) size, the distribution of tesseral sizes broadened and shifted toward larger tesserae (Figure 11). Compared to the 24 cm *Rhina* jaw, for example, tesserae in the 35 cm *Rhina* jaw were nearly 40% larger (from 456.8 ± 120.3 µm to 632.3 ± 199.7 µm: mean ± standard deviation; Supplementary Table S2). Similarly, in *Rhynchobatus*, tesserae showed almost a twofold average size increase from the smallest animal to the largest, ranging from 606.6 ± 186.4 µm in the 11 cm jaw to 1086.4 ± 338.6 µm in the 28 cm jaw (Supplementary Table S2). The largest tesserae recorded in both species (2315.4 µm in *Rhina*, 4504.6 µm in *Rhynchobatus*) represent the tesserae we observed beneath the teeth, fused together into mineralized sheets (*see* below; Figures 9, 10).

In jaw cross sections, tesserae showed differences in orientation and connectivity within and among the different tesseral layers (Figures 3B,C, 4B,C and Supplementary Figures S1B,C, S2B,C). In the outermost layer, the contacts between adjacent columnar tesserae appeared tightest peripherally (nearest the perichondrium), especially compared to the looser packing of scree tesserae in deeper layers. Throughout the jaw cortex, minute projections were regularly seen bridging adjacent tesserae (see insets in Figures 3B, 4B); these were often very bright in tomographic slices, indicating they are the hypermineralized “spokes” known to reinforce intertesseral joints in other species (Seidel et al., 2016, 2019, 2020). In *Rhina* and *Rhynchobatus*, multiple spokes could often be seen spanning a single joint space, particularly in the perichondral layer (e.g. Figures 3B, 4B insets). Spokes appeared irregularly distributed in both taxa (i.e. not visible between every tessera in every slice) and were more numerous in *Rhynchobatus*. Spokes were not as apparent in the smallest individual sampled (*Rhynchobatus*, Supplementary Figures 1B,C), nor in the largest (*Rhina*, Supplementary Figures S2B,C). It is unclear from the current data whether this was a function of the spokes being less prevalent (as in younger animals; Seidel et al., 2016), or their presence being masked by the lower resolution resulting from the large field of view needed to scan the largest specimen (Supplementary Table S1).

The density of tesserae (based on gray values, calculated from the attenuation coefficient) appeared generally consistent within datasets, similar to tooth dentin and skin denticles, but less mineral-dense than tooth enameloid (e.g. Figures 3B,C, 4B,C). Whereas smaller specimens of *Rhina* and *Rhynchobatus* showed more consistent density contrast in all tesseral layers (Figures 3B,C and Supplementary Figures S1B,C), in the largest *Rhina* (Supplementary Figures S2B,C) and especially the largest *Rhynchobatus* (Figures 4B,C), tesserae had a visibly lower density in areas directly below the dentition, implying a lower degree of mineralization. In these regions in *Rhynchobatus* and in the very large *Rhina*, tesserae at the oral surface were large and often partially fused together into irregular mineralized masses (e.g. Supplementary Figure S2), in extreme cases resulting in the tesseral pattern being obliterated and replaced by a nearly homogeneous tissue of lower mineral density (Figures 4B,C).

#### Trabeculae and trabecular tesserae

Trabeculae (reinforcing struts passing through the core of the jaws) were present in both species (Figures 3, 4, 9D,G, 10D,G, 12 and Supplementary Figures 1B,C, 2B,C, Supplementary Movies S1–S3). Trabeculae in *Rhynchobatus* were tessellated tubes with walls one tesseral layer thick (Figures 4B,C, 10D,G). In contrast, in *Rhina*, trabeculae often appeared only partially mineralized (Figures 3B,C), as darker gray (less-mineralized) tubes studded with tesserae. It was impossible to determine, however, the degree to which these trabeculae might have collapsed, lost tesserae, or become degraded as these museum specimens were dried and the internal cartilage pulled away during dehydration.

In both species, trabeculae typically ran between the labial and lingual cortical surfaces (e.g. Figures 3C,D, 4C,D, 12), but with some exceptions (*see* below). The internal canals formed by trabeculae were often open at each end, communicating to the perichondrium through visible pores, particularly in *Rhina* (e.g. Figures 9C,H and Supplementary Figure 2G). In *Rhina*, trabeculae represented 13.6% and 12.2% of the volume of the mineralized tissue in the upper and lower jaws, respectively (Figure 12A and Supplementary Table S3). In contrast, trabeculae were far less dominant in *Rhynchobatus*, representing only 5.2% and 4.7% of the upper and lower jaw volumes, respectively (Figure 12B and Supplementary Table S3). The abundance of trabeculae in *Rhina* was particularly apparent in the distal jaw region associated with teeth, where trabeculae dominated the interior of the jaw, orientated in numerous directions (oral-aboral, labio-lingual; Figures 9D,G, 12A), and even appearing to course parallel to the cortex of the oral surface (i.e. in the proximo-distal direction) in some areas (e.g. circular trabecular sections in Figures 3B, 12A [trab*] and Supplementary Movies S1–S3). In contrast, trabeculae in *Rhynchobatus*, tended to be oriented labio-lingually, although some proximo-distal trabeculae were also observed (Figures 4B,C, 10D,G, 12B).

#### Teeth

Our tomographic slices provided clear views of the process of tooth development in both species (Figures 3B,C, 4B,C and Supplementary Figures S1B,C, 2B,C). New teeth arose at the lingual side of the jaw, appearing in our micro-CT data as hollow shells covered by dense enameloid caps and anchored to the dental lamina by thin dentin bases. As the teeth developed and progressed labially, the enameloid layers became thicker and the interior pulp cavities were gradually filled with mineralized dentin, before the teeth reached their functional positions. In some sections, the enameloid layer of post-functional teeth appeared slightly thinner, suggesting some wear. The enameloid layer was thicker on average in *Rhina* (Figures 3B,C versus Figures 4B,C). Both species showed surface ornamentation on their teeth: a single latero-medial ridge on each tooth crown of *Rhynchobatus* (occasionally with smaller secondary ridges visible in cross section; inset in Figure 4B), in contrast to a series of labio-lingual ridges in *Rhina* (typically eight per tooth; inset in Figure 3C). These ornamentations were sculpted predominantly from enameloid, with only a slight associated undulation of the enameloid-dentin junction. In cross-section, points of direct labio-lingual contact were visible between teeth in a tooth file (labio-lingual series; Figures 3B,C, 4B,C), the teeth touching at only a single point in *Rhynchobatus*, but effectively interlocking in *Rhina*. In both species, points of contact always appeared to be between enameloid-coated tooth regions, not where dentin was exposed.

Teeth in both taxa were largest and the dentitions thickest where associated with the meta-teeth: at the symphysis on the lower jaw and parasymphyseally on the upper jaw in both species, with additional less pronounced meta-teeth also present parasymphyseally on the lower jaw in *Rhina* (Figures 3A,B, 4A,B, 5A,B, 6A,B). Conversely, the concave regions of the undulating dentition (i.e. those that “received” the meta-teeth) were covered by comparatively small teeth. The largest teeth (those forming the meta-teeth) were also slightly more bulbous, whereas the teeth associated with concave regions of the jaws and the proximal end of the dentition were slightly flatter (Figures 3, 4). In jaws of similar size (e.g. Figures 5A,B, 6A,B), the dentition of *Rhina* was more robust (typically ≥1 mm thicker).

### Myliobatiformes (*Aetobatus*), Rajiformes (*Raja*)

The dentition of the durophagous stingray *Aetobatus* (Myliobatiformes) consists of a single symphyseal file of elongate teeth in both the upper and lower jaws (Figure 7). *Aetobatus* teeth were much thicker than the jaw’s cortex (Figures 7C,D), which bore several tesseral layers in the upper jaw (four to five layers, in some areas), but fewer in the lower jaw (Figures 7B1,B2 and Supplementary Movie S4). As with *Rhina* and *Rhynchobatus*, the regions with the most cortical tesseral layers were directly beneath the functional teeth, although the number of cortical tesseral layers was generally far fewer than in the rhinopristiform taxa. *Aetobatus* tesserae ranged in size from ∼100 to 500 μm, with no irregularly-shaped tesserae observed (e.g. the perichondral columnar tesserae of *Rhina* and *Rhynchobatus*). The teeth were capped by a thin layer of enameloid (<300 µm thick), with the bulk of the tooth thickness provided by dentin. As with *Rhina* and *Rhynchobatus*, gray values of micro-CT data show that internal teeth tissues gradually mineralize during development, with the dentin of the newest forming teeth being poorly mineralized, compared to the more highly mineralized functional teeth. In *Aetobatus*, however, older teeth were considerably worn, being only ∼10% their starting height (apparently due to removal of both enameloid and dentin). Trabeculae were far more numerous than in either *Rhina* or *Rhynchobatus*, representing 37.1% and 33.2% of the mineralized tissue volume of the upper and lower jaws, respectively. *Aetobatus* trabeculae occurred throughout the jaw (Figures 7B1,B2 and Supplementary Movies S1–S6), running primarily from the oral to aboral jaw surfaces. Trabeculae in this species were also more irregularly-shaped than in either rhinopristiform species, appearing to branch and anastomose toward the oral jaw surface. Although trabeculae were tessellated near the oral surface of the jaw, aborally, trabeculae appeared to lack tesserae (as in *Rhina*, described above).

In *Raja*, the teeth were small and pointed with a thin enameloid cover (Figure 8; a male specimen, in females the teeth are also small, but flatter and rounded; Underwood et al., 2015), and of similar thickness to the jaw cortex (Figure 10D). Tesserae in *Raja* range between 400 and 900 μm in size, having the regular prismatic shape previously described for batoid fishes (Seidel et al., 2020). In cross section, the cortical tesserae appeared thicker than those of *Aetobatus*, although only a single layer was present in *Raja*, except beneath the dentition, where additional layers of smaller tesserae occurred (Figure 8C). Trabeculae were absent, although what appeared to be an unmineralized or poorly mineralized strut was visible in one section (Figure 8C2).

## Discussion

The rhinopristiform species investigated here demonstrate multiple strategies for jaw reinforcement against a hard prey diet, indicating species- and order-level differences, while also illustrating that the accepted anatomical correlates of durophagy in elasmobranchs (e.g. Summers, 2000; Dean et al., 2006; Seidel et al., 2021) are more varied and modular than previously appreciated.

### Tesserae

Although closely related, *Rhina* and *Rhynchobatus* exhibited clear differences in overall thickness of the jaw cortex; in degrees of mineralization in tesserae associated with the jaw cartilage; in size, shape and arrangement of individual tesserae; in number, location, orientation and degree of mineralization of trabeculae; and in thickness of enameloid and the functional dentition (Figures 1, 3, 4). Both species, however, shared an obvious reinforcement of dental and skeletal features in association with the occlusal regions of the jaw. In both taxa, larger jaws exhibit greater cortical thickness distally along the jaw at the oral surface (Figures 5, 6). In *Rhynchobatus*, the cortical thickness below the dentition (in the form of multiple layers of tesserae) can be more than double that of *Rhina* (Figures 3–6, 9, 10). This thickening, however, is not the result of *Rhynchobatus* possessing more layers of tesserae, but rather having larger tesserae overall (Figures 9,10 and Supplementary Table S2). In contrast, in *Rhina*, local cortical thickening is accomplished through assemblies of smaller tesserae arranged into a thickened, disorganized scree (Figure 3).

In the rhinopristiform species we examined, dentition-associated cortical thickening apparently becomes more pronounced with age, suggestive of an adaptive response. Tessellated cartilage is believed to have limited to no remodeling ability (Clement, 1992; Dean et al., 2015; Marconi et al., 2020) and therefore the mineralized layer can only grow through addition of new material (Dean et al., 2009, 2017; Seidel et al., 2016). Across the *Rhina* and *Rhynchobatus* specimens examined, the distribution of tesseral sizes broadens and shifts in the direction of increasingly larger tesserae as animals increase in size (Figure 11 and Supplementary Table S2). These data therefore provide the first broadscale support of a tessellated cartilage growth hypothesis based previously only on 2D slices from limited numbers of tesserae, arguing that skeletal growth is accomplished (at least in part) by tesserae increasing in size with age (Dean et al., 2009; Seidel et al., 2016, 2019). Growth of the cortex could also be a function of the addition of new (small) tesserae interstitially in the tesseral layer; however, the rightward shifting of our tesseral size distributions away from smaller tesserae argues this mechanism is either not occurring or is comparatively uncommon.

Our data also suggest that tesserae may not be growing at uniform rates. In *Rhynchobatus*, tesseral size appears to become more heterogeneous with age: in comparison with the smaller individuals, tesserae size in the larger *Rhynchobatus* specimen is more variable (Figures 11C, D and Supplementary FigureS1). In both *Rhynchobatus* and *Rhina*, the perichondral (outermost) tesseral layers involve massive tesserae with striking columnar morphologies (Figures 3, 4, 9, 10). Such high-aspect-ratio tesserae have been likened by Maisey et al. (2021) to the “voussoir” stones used by stonemasons to build the curved portions of archways; similarly, most images of voussoir tesserae suggesting they have a quite local distribution, associated with skeletal ridges and the margins of foramina in tessellated cartilage (Seidel et al., 2016; Maisey et al., 2021). Our data, however, show columnar tesserae to be the primary tesseral morphology of the perichondral tesseral layer in the jaws of durophagous *Rhynchobatus* and *Rhina*, not only limited to curved regions. This argues that these large tesserae may be important for resisting high mechanical loads, as well as for constructing strongly curved surfaces (i.e. regions with small radii of curvature). Also, accepting that tesserae increase in size with age, the larger size of voussoir tesserae could indicate that the perichondral tesseral layer is the oldest in the jaw skeleton. This would suggest that the inner (chondral) layers of smaller scree tesserae developed after perichondral tesserae, within the unmineralized cartilage (Maisey et al., 2021), their disorganization perhaps suggesting a more rapid development in response to increased feeding stresses as the animals grew. Alternatively, columnar tesserae might be larger due to more rapid growth (e.g. in response to high mechanical loads) and scree layering could simply be a constructional constraint of building highly curved cross-sections from individual brick-like elements (tesserae). The hypothesis that larger tesserae and/or a thicker jaw cortex develop in response to load (or at least are involved in resisting higher loads) is also supported by the occurrence of thicker tesseral layers below the dentition (in *Rhina* and *Rhynchobatus*, but also in *Aetobatus* and *Raja*) and associated with the jaw joint (*Rhina*, *Rhynchobatus*, *Raja*) (Figures 5C,D, 6C,D, 7C,D, 8D). The presence of tessellated sesamoid cartilages beneath the symphyseal meta-teeth in both rhinopristiform species is further suggestive of high local mechanical stresses (Sarin et al., 1999; Fontenelle et al., 2018).

The largest tesserae we measured were irregular and less-mineralized, observed in the oral cortex of larger *Rhina* and especially *Rhynchobatus* specimens (Figures 9, 10 and Supplementary Figure S2, Supplementary Table S2). We believe these to be the product of fusions of individual tesserae. It is possible that these structures instead represent groups of tesserae with particularly narrow gaps between them (i.e. beyond the resolutions of our scans), but we find this unlikely, given that individual tesserae were successfully resolved in all other scan regions. The irregular shape of these tesserae is a significant departure from the polygonal (or at least symmetrical) tesserae of other batoid taxa (e.g. *Urobatis*, *Aetobatus*, *Raja*; Seidel et al., 2016, 2020, 2021; Atake and Eames, 2021) and of the smaller *Rhina* and *Rhynchobatus* sampled here (Figures 9, 10 and Supplementary Figure S2). Similar irregular tesserae, fused together at their perichondral surfaces, have been observed in the jaw cortices of other species, either in healthy tissues (Maisey, 2013; Maisey et al., 2021) or associated with a callus-building damage response (Dean et al., 2017). Maisey (2013) hypothesized this morphology represented a breakdown of the inhibition of mineralization in the joints between tesserae, noting that this morphology did not always occur in regions of high stress. The similar irregular mineralization we observed in larger *Rhina* and *Rhynchobatus* could therefore be associated with age, however, the lower level of mineralization in these tesserae is more difficult to explain. Perhaps these morphologies indicate tesserae with an especially high organic content (e.g. particularly large Sharpey’s fibers; Seidel et al., 2017), as might be needed where the fibrous dental ligament is anchored into the skeleton.

### Trabeculae

**FIGURE 13:**
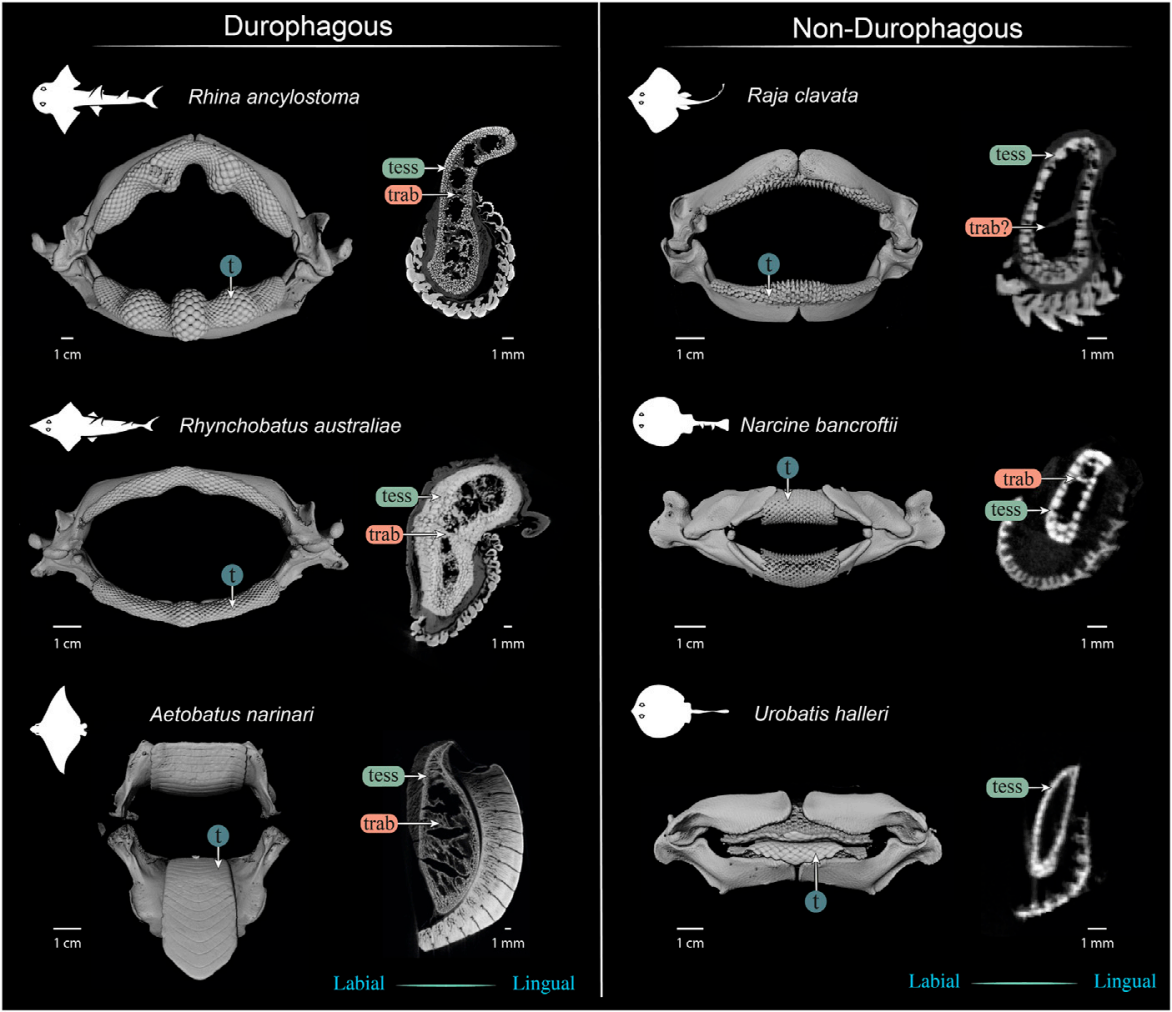
Comparison of jaws and upper jaw cross sections of durophagous (left) and non-durophagous batoid species (right). The durophagous batoid jaws shown here are highly “structured,” involving large tesserae and/or numerous tesseral layers reinforcing the cortex, and well-defined tessellated trabeculae (trab), but the preponderance of these characters varies among the species shown here. The non-durophagous jaws are simpler in their cross-sectional shape and sheathed mostly in a single layer of tesserae, but some regions bear features previously associated only with durophagous taxa. For example, multiple tesseral layers are visible in the jaw cortex at the oral ends of all jaw cross sections (beneath the teeth) and sparse load-leading trabeculae are visible in the jaws of *Narcine bancroftii* and perhaps in *Raja clavata* (trab?), that of the latter albeit not surrounded by tesserae. In contrast, the jaws of *Urobatis halleri* were entirely devoid of trabeculae and bore a comparatively thin cortex. The *Narcine* and *Urobatis* scans are from unregistered specimens, scanned for other studies.

In addition to surface reinforcements of the skeleton, trabeculae were present in the jaws of all durophagous species examined (*Rhynchobatus*, *Rhina* and *Aetobatus*), but absent in *Raja* and also *Urobatis* (*see* summary diagram, Figure 13). In both *Rhynchobatus* and *Rhina*, trabeculae were simple, relatively linear tubes (Figure 12). Whereas trabeculae were typically covered by a single layer of stout tesserae in *Rhynchobatus*, they apparently bore only a patchy tessellated covering in some regions in *Rhina*, although trabeculae in this species were roughly three times as densely packed as in *Rhynchobatus* (Figures 3, 4 and Supplementary Movies S1–S3). The patchy tessellation of *Rhina* trabeculae is similar to the trabeculae of the *Aetobatus* jaw we examined (Figure 7 and Figures 9, 10 and Supplementary Movie S4), where trabeculae appeared to lack tesserae at their aboral ends (but *see* caveat above about the dried nature of the specimens). In contrast to the rhinopristiform species, the trabeculae of *Aetobatus* were hierarchically branched and far more densely populated.

The orientations of trabeculae in all three durophagous species suggest differences in loading patterns associated with feeding. “Load leading” trabeculae (oriented in-line with the bite force; Dean et al., 2006) were situated beneath the dentition in both the upper and lower jaw cartilages of *Rhina* and *Aetobatus*; the durophagous diets of these species can explain the need for such support, preventing collapse of the jaw cortex when exerting the force necessary to fracture invertebrate exoskeletons. The irregular and branching nature of *Aetobatus* trabeculae may compensate for their comparatively thin walls, but may also indicate this species employs more diverse (e.g. multi-axial) prey-processing behaviors. Conversely, the apparent lack of load-leading trabeculae in *Rhynchobatus* may be accounted for by the species’ particularly robust jaw cortex. Both *Rhina* and *Rhynchobatus* also exhibited trabeculae oriented perpendicular to the direction of bite force, “truss trabeculae” bridging the labial and lingual jaw cortices (Dean et al., 2006), particularly in narrow regions of the jaw cross-section (Figures 3B,C, 4B,C, 9D,G, 10D,G). As with horizontal tie-bars used to brace and strengthen brickwork walls (e.g. Spina et al., 2004; Zielińska, 2017), truss trabeculae likely help maintain the jaw shape during biting by preventing structural buckling (Dean et al., 2006). Whereas truss trabeculae were largely found in the mid-shaft (parasymphyseal regions) of the jaw in both rhinopristiform species, load-leading trabeculae were absent in *Rhynchobatus* and concentrated closer to the symphysis in *Rhina*, suggesting some local division of labor in the jaws, perhaps associated with the undulating dentitions of these species. A third class of trabeculae was observed in both rhinopristiform species, perpendicular to both load-leading and truss trabeculae, in line with the long (proximo-distal) axis of the jaws (trab* in Figure 3B and Figures 9, 10 and Supplementary Movies S1–S6). To our knowledge, such trabeculae have not been previously described. Although their role is unclear, their orientation could argue they provide either additional structural support (e.g. like longitudinal bars in steel-reinforced concrete beams) or even a non-mechanical function (e.g. nutrient transport). Mapping the full trabecular network in hydrated samples would help to clarify the true diversity of their functional roles.

### Teeth

The teeth of durophagous species are in a battle of contact and fracture mechanics with their prey, working to cause damage to prey shells and exoskeletons, but without tooth materials being damaged in return (Lucas et al., 2008; Amini et al., 2020). The nature of contact between tooth and prey is a deciding factor in which surface is damaged (e.g. tooth or shell) with the radius of curvature of the contacting tooth being hugely important to the type of damage caused (Lucas et al., 2008; Crofts and Summers, 2014). For a given prey item, as teeth become flatter (i.e. with larger radius of curvature), they will tend to cause more far-field damage (at a distance from the contact point) and through-thickness shell failure (Lucas et al., 2008). These are more destructive damage modes than produced by near-contact stresses (Lucas et al., 2008), although smaller indenters, like pointed tooth cusps, can be quite effective in initiating cracks (Crofts and Summers, 2014). Furthermore, for a given tooth diameter and prey item, flat and domed teeth require less force than concave ones to initiate crack propagation in prey exoskeletons, although this is conversely also dependent on the size of the prey item, relative to the concavity (Crofts and Summers, 2014).

The distinctive curved dentitions of *Rhina* and *Rhynchobatus* take advantage of the geometric factors facilitating prey fracture. Although containing smaller teeth with far smaller radii of curvature than myliobatiform stingray dentitions (this study; Kolmann et al., 2015a), the bulbous meta-teeth of the rhinopristiform species, particularly pronounced in *Rhina*, massively increase the radius of curvature of contact with prey items, creating larger “effective cusps” more suited for pulverizing shells. Additionally, although the cross-sectional shape of myliobatiform jaws has been shown to have little impact on shell crushing performance (Kolmann et al., 2015a)*,* the undulating oral jaw surfaces of *Rhina* and *Rhynchobatus* provide a sculptured loading platform for prey items, the biological equivalent of an engineering three-point bending rig, allowing the meta-teeth to act as large, local stress concentrators.

Compared to most other examined batoid fishes, *Rhina* and *Rhynchobatus* have thicker enameloid, comprising a compact outer layer of randomly-orientated crystallites and an inner parallel-organized layer, with crystallites orientated perpendicular to the tooth surface (Enault et al., 2013; Manzanares et al., 2016). This thickened microstructure is believed to impart compression resistance to the enameloid (Gillis and Donoghue, 2006; Enault et al., 2013; Amini et al., 2020). Coupled with teeth being interlocked, and tooth ridges and meta-teeth surely enhancing the grip on prey (particularly in *Rhina*), these dental features create a stabilized platform for crushing behaviors. The comparatively large teeth that comprise meta-teeth (Figures 3–6) and the broken cusps observed in two of our specimens’ meta-teeth (asterisk in Figures 3A, 5A) are perhaps indications of the exceptional local stresses generated in these areas. The robust and self-supporting nature of *Rhina* and *Rhynchobatus* dentitions is also demonstrated by their spanning of the jaw symphyses, which we show are unfused and flanked by the distal jaw tips, which have surprisingly thin-walled cortices (Figures 3, 4). This is in stark contrast to the jaws of *Aetobatus*, where the jaw halves are fused at the midline into stout, single elements (Figure 7). In *Rhina* and *Rhynchobatus*, the dentitions (and perhaps, sesamoid cartilages) must therefore also act as structural girders to support the jaws at the midline, a function not typically attributed to teeth.

### Toward a synthesis of elasmobranch durophagy

It is clear that “durophagy” is too general a descriptor for the diverse diets and morphologies of elasmobranch fishes typically placed in this category. For example, whereas the diet of *Aetobatus* is dominated by hard-shelled molluscs (Schluessel et al., 2010), *Rhina* is known to feed on fish, prawns, and cephalopods, in addition to crabs and bivalves (Compagno and Last, 1999; Raje, 2006). The stomach contents of *Rhynchobatus*, by comparison, indicate a predominantly shrimp-based diet, with fish and crabs eaten only by larger individuals (Nasir, 2000; Abdurahiman et al., 2010), while a recent study, based on spines embedded in the jaws (*see* also Figure 10F), suggested that *Rhynchobatus* may also prey on smaller stingrays (Dean et al., 2017). Anyone who has eaten seafood knows that the strategies for crushing mollusc shells differ considerably from those for processing crustaceans like shrimp. Yet to date, no experimental study has looked broadly enough at durophagous feeding anatomy and performance in elasmobranchs to resolve more subtle ecomorphological connections.

Our data, combined with anatomical data from previous works (e.g. Summers, 2000; Kolmann et al., 2015a; Rutledge et al., 2019) begin to frame a more holistic view of elasmobranch durophagy, mapping out a suite of modular morphological characters, which can be diversely combined to reinforce against extreme feeding loads. A comparison of several of the batoid fishes most-studied in anatomical research (Figure 13) illustrates the potential interrelationships of morphological characters, underlining differences in diet, tesseral shape and layering, trabecular presence and orientation, and dentition (including enameloid thickness and surface ornamentation). Cortical tesserae, for example, can vary in their size and shape, and in how orderly and numerous their layers are. Thicker cortices are certainly associated with regions of high load, even in non-durophagous species, but this can be variously achieved, by employing massive tesserae (e.g. perichondral columnar tesserae), numerous tesseral layers, or both. Such reinforcements seem to come with departures from the “typical” polygonal tesseral forms, in perichondral and chondral tesseral layers. Symphyseal fusion, more massive and flatter teeth, and teeth interacting to form superstructures (e.g. meta-teeth) also occur in species experiencing high feeding stresses. Trabeculae, also present in their “truss” (labio-lingual) orientation in non-durophagous species, occur in far higher densities and with telltale load-leading (oral-aboral) alignment in species with molluscs in their diets.

These characters can all be involved in jaw reinforcement for durophagy, yet they appear to trade-off in their preponderance: *Rhynchobatus* possesses a thicker jaw cortex (comprised of fewer, but more massive tesserae, fused into mineralized concretions beneath the teeth), but no load-leading trabeculae, an unfused symphysis, and shorter teeth with a low degree of interlocking and tooth superstructuring (i.e. assembly into meta-teeth). *Rhina*’s unfused symphysis and thinner jaw cortex (from smaller, but more numerous tesserae), is compensated for by thick-walled trabeculae (albeit in some regions only partially tessellated), oriented in line with loading and a more robust dentition, with thicker teeth, thicker enameloid and massive meta-teeth. The jaws of *Aetobatus* have a comparatively thin cortex (comprised of several layers of thin, platelike tesserae), but are filled with a dense stand of load-leading trabeculae (thin-walled, but branching), the jaws fused at the symphysis, and the extremely thick and interlocking teeth creating a monolithic dental plate.

Previous comparisons of durophagous shark and ray species have suggested that elasmobranch lineages invest to different degrees in shape-vs. structure-vs. material-solutions for jaw reinforcement (Summers et al., 2004; Huie et al., 2022). Including our data, these observations of varied reinforcement strategies argue that durophagy is an interestingly multivariate problem in elasmobranchs with diverse solutions, yet the pressures driving the evolution of the different character combinations are unclear. The key to clarifying this is multi-disciplinary: by examining and integrating feeding behavior and mechanics, gut content, anatomical, and biological materials data, we can better resolve the factors that have shaped extreme feeding modes and determine their links to phylogeny, prey co-evolution and biogeography.

## Data Availability

Renderings of the primary microCT datasets are provided in annotated videos in the Supplementary Material. Additional microCT data is available from the authors by request.
