# Supplementary figures and images for: Bricks, trusses and superstructures: Strategies for skeletal reinforcement in batoid fishes (rays and skates)
Source: Front Cell Dev Biol. 2022 Oct 12;10:932341. doi: 10.3389/fcell.2022.932341 (PMC9604235; doi:10.3389/fcell.2022.932341)

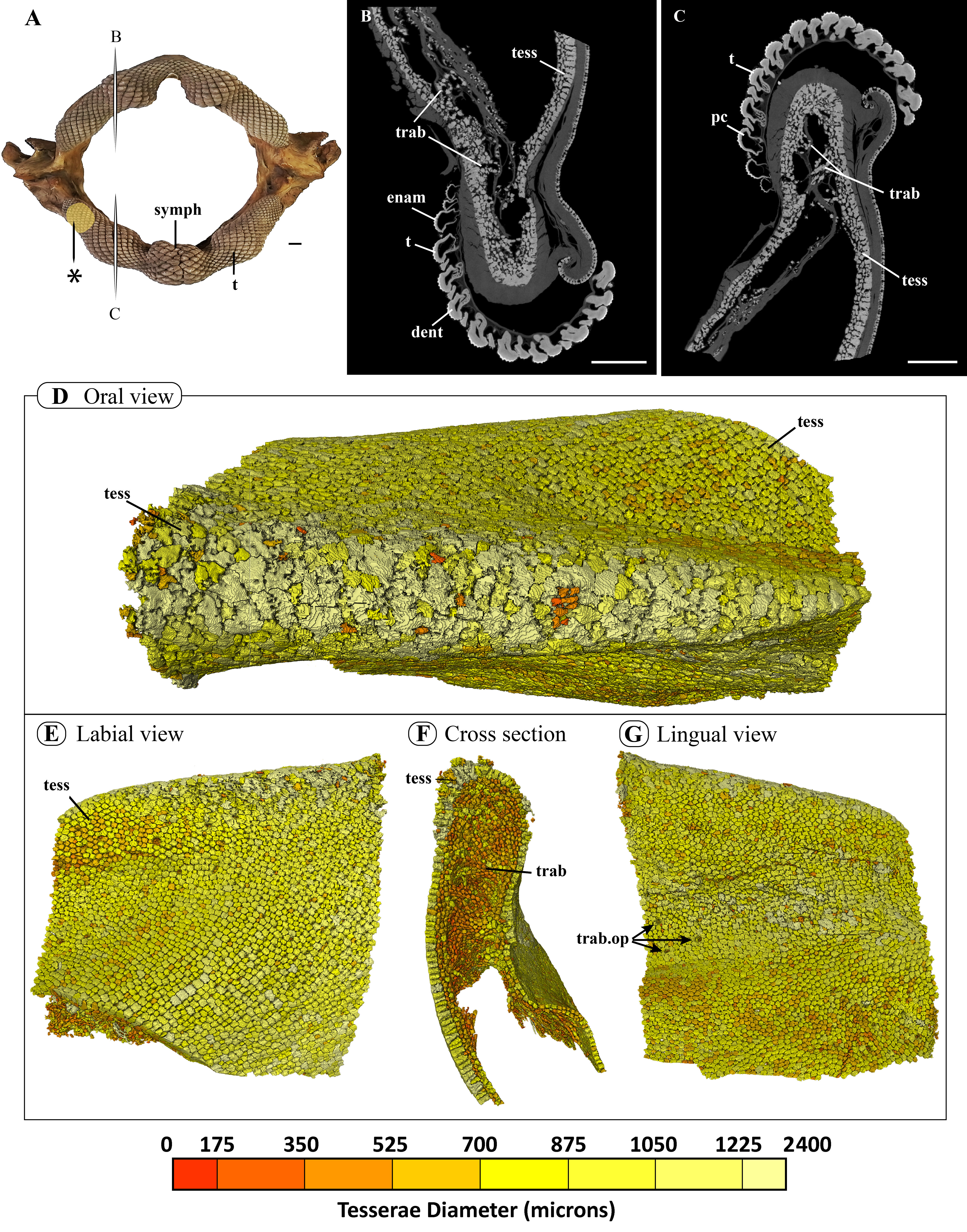

Supplement: Supplementary file 1 [file Image3.JPEG]

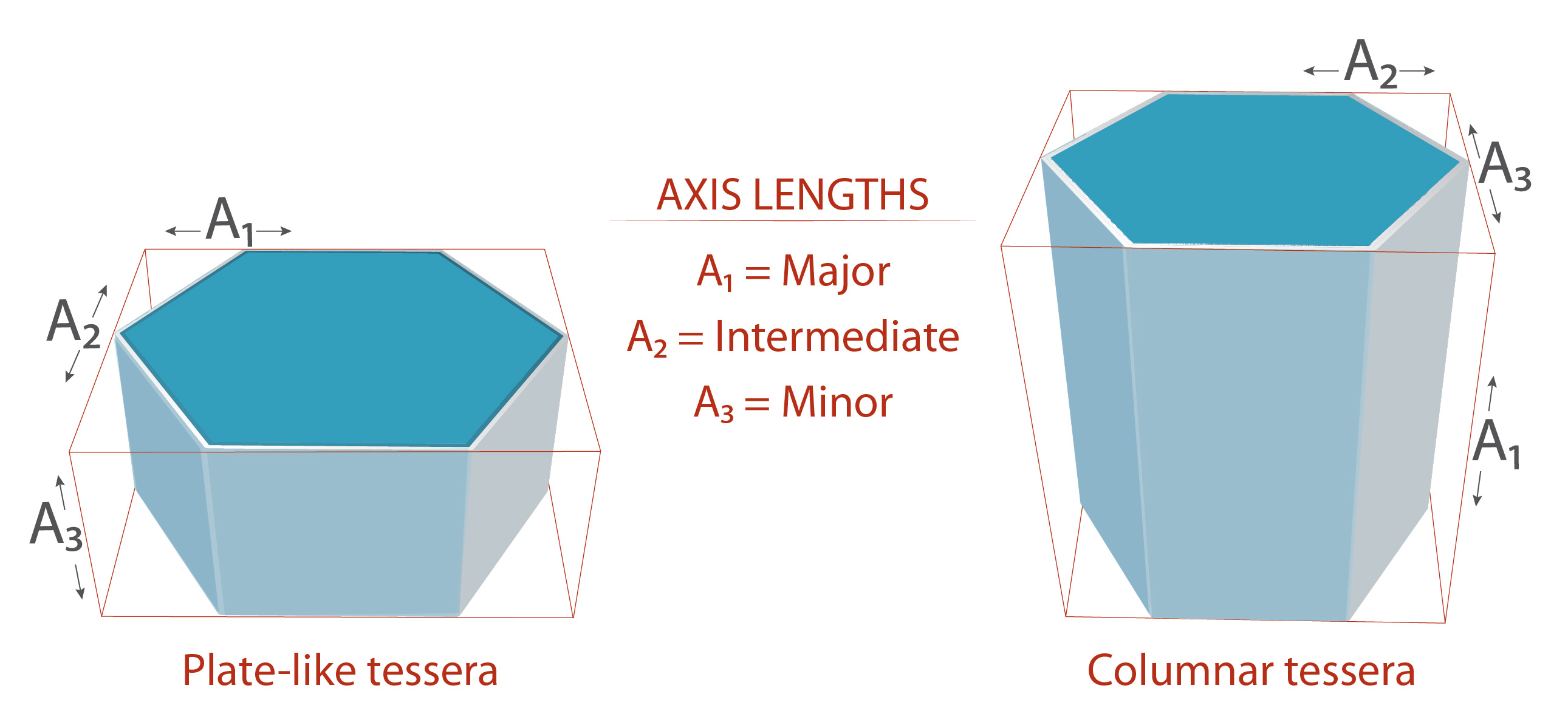

Supplement: Supplementary file 4 [file Image1.JPEG]

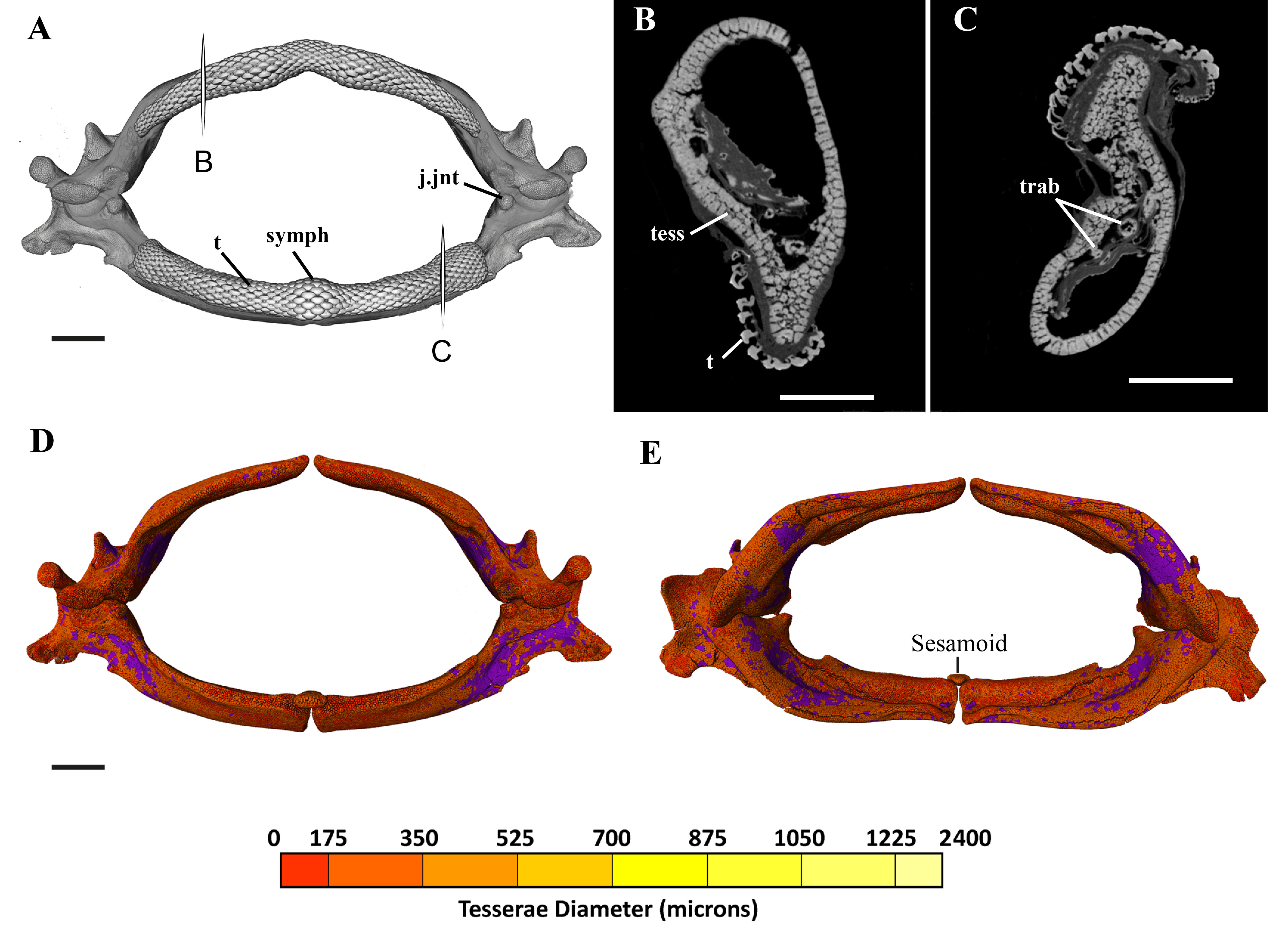

Supplement: Supplementary file 5 [file Image2.JPEG]
