# Supplementary material for: Bricks, trusses and superstructures: Strategies for skeletal reinforcement in batoid fishes (rays and skates)
Source: Front Cell Dev Biol. 2022 Oct 12;10:932341. doi: 10.3389/fcell.2022.932341 (PMC9604235; doi:10.3389/fcell.2022.932341)
Supplement: Supplementary file 3 [file Table1.DOCX]

| Museum accession | | | Regions analyzed + voxel sizes | | Analysis | |
| --- | --- | --- | --- | --- | --- | --- |
| Species | number | Jaw width | |  | |  |
| *Rhina ancylostoma* | BMNH 2015.1.25.5 | ~ 24 cm | | Full jaw (121.68 µm) | | Dental + cortex thickness |
|  |  |  |  |  |  | Trabeculae morphology + % |
|  |  |  |  | ROI: Lower left proximal (26.37 µm) | | Tesserae size analysis |
|  |  |  |  | ROI: Upper left proximal (26.37 µm) | | Tesserae size analysis |
| *Rhina ancylostoma* | BMNH 2014.11.11.1 | ~ 35 cm | | ROI: Lower right proximal (32.97 µm) | | Tesserae size analysis |
|  |  |  |  |  |  | Trabeculae morphology + % |
| *Rhynchobatus* sp | Unregistered specimen | ~ 11 cm | | Full (36.33 µm) | | Tesserae size analysis |
|  |  |  |  |  |  | Trabeculae morphology + % |
| *Rhynchobatus australiae*? | Unregistered specimen | ~ 18 cm | | Full (66.18 µm) | | Tesserae size analysis |
| *Rhynchobatus* sp | NHMUK PV P4808 | ~ 25 cm | | ROI: Lower right (36.27 µm) | | Tesserae size analysis |
| *Rhynchobatus australiae* | BMNH 2017.7.11.1 | ~ 28 cm | | Right side of the jaw (84.41 µm) | | Dental + cortex thickness |
|  |  |  |  |  |  | Trabeculae morphology + % |
|  |  |  |  | ROI: Lower left proximal (38.99 µm) | | Tesserae size analysis |
|  |  |  |  | ROI: Upper left proximal (28.43 µm) | | Tesserae size analysis |
| *Aetobatus ex. gr. narinari* | BMNH 2015.1.25.4 | ~ 7.5 cm | | Full jaw (38.70 µm) | | Dental + cortex thickness |
|  |  |  |  |  |  | Tesserae size analysis |
|  |  |  |  |  |  | Trabeculae % |
| *Raja clavata* | BMNH 2015.1.25.2 | ~ 9 cm | | Full jaw (55.70 µm) | | Dental + cortex thickness |
|  |  |  |  |  |  | Tesserae size analysis |

**Table S1.** Specimens and regions of interest used in the study, micro-CT parameters, and analyses performed.

**Table S2.** Descriptive statistics for tesserae size for *Rhina,* *Rhynchobatus, Raja clavata* and *Aetobatus ex. gr. narinari* specimens analyzed.

| **Tesserae size**  **(µm**) | ***Rhina ancylostoma*** | | ***Rhynchobatus* spp** | | | | ***Raja clavata*** | ***Aetobatus ex. gr. narinari*** |
| --- | --- | --- | --- | --- | --- | --- | --- | --- |
|  | **24 cm** | **35 cm*** | **11 cm** | **18 cm** | **25 cm*** | **28 cm*** | **9 cm*** | **7.5 cm*** |
| Min | 28.8 | 212.9 | 246.8 | 172.7 | 227.4 | 288.3 | 104.0 | 109.5 |
| Median | 439.2 | 607.2 | 566.5 | 720.6 | 808.9 | 1027.8 | 373.4 | 302.9 |
| Mean | 456.7 | 632.3 | 606.6 | 705.3 | 824.8 | 1086.4 | 369.1 | 306.7 |
| Max | 1357.7 | 2315.4 | 1898.3 | 1638.9 | 1891.4 | 4504.6 | 823.6 | 541.9 |
| Stdev | 120.3 | 199.6 | 186.3 | 189.8 | 250.8 | 338.5 | 163.1 | 71.1 |

**Table S3.** Volume percentages for trabeculae and non-trabecular mineralized tissue (i.e. cortical tesserae) for *Rhina*, *Rhynchobatus*, and *Aetobatus* specimens. Percentages are relative to total mineralized skeletal tissue (i.e. trabeculae + cortical tesserae).

| **Skeletal element (%)** | ***Rhina ancylostoma* (24 cm)** | | ***Rhynchobatus australiae* (28 cm)** | | ***Aetobatus ex. gr. narinari* (7.5 cm)** | |
| --- | --- | --- | --- | --- | --- | --- |
|  | **Upper jaw** | **Lower jaw** | **Upper jaw** | **Lower jaw** | **Upper jaw** | **Lower jaw** |
| Trabeculae | 13.53 | 12.17 | 5.25 | 4.64 | 37.06 | 33.18 |
| Non-trabecular mineralized tissue | 86.46 | 87.82 | 94.74 | 95.35 | 62.93 | 66.81 |
